# Supplementary material for: A new tool for evaluating health equity in academic journals; the Diversity Factor
Source: PLOS Glob Public Health. 2023 Aug 14;3(8):e0002252. doi: 10.1371/journal.pgph.0002252 (PMC10424852; doi:10.1371/journal.pgph.0002252)
Supplement: Attachment — Submitted filename: PGPH-D-23-00134_R1.pdf [file pgph.0002252.s002.pdf]

PLOS Global Public Health

A new tool for evaluating health equity in academic journals; the Diversity Factor

--Manuscript Draft--

Manuscript Number:	PGPH-D-23-00134R1
Article Type:	Research Article
Full Title:	A new tool for evaluating health equity in academic journals; the Diversity Factor
Short Title:	The Diversity Factor
Corresponding Author:	Jack Gallifant<br>Guy's and St Thomas' NHS Foundation Trust<br>London, UNITED KINGDOM
Order of Authors:	Jack Gallifant
	Joe Zhang
	Stephen Whebell
	Justin Quion
	Braiam Escobar
	Judy Gichoya
	Karen Herrera
	Ruxana Jina
	Swathikan Chidambaram
	Abha Mehndiratta
	Richard Kimera
	Alvin Marcelo
	Portia Grace Fernandez-Marcelo
	Juan Sebastian Osorio
	Cleva Villanueva
	Lama Nazer
	Irene Dankwa-Mullan
	Leo Anthony Celi
Keywords:	
Abstract:	Current methods to evaluate a journal's impact rely on the downstream citation mapping used to generate the Impact Factor. This approach is a fragile metric prone to being skewed by outlier values and does not speak to a researcher’s contribution to furthering health outcomes for all populations. Therefore, we developed the Diversity Factor to fulfil this need and comprised four key elements: dataset properties, author country, author gender and departmental affiliation. Herein, we develop the methodology to reproducibly calculate these elements, evaluate the current landscape and publish the findings on a freely available website that enables further evaluation.The OpenAlex database was used to extract the metadata of all papers published from 2000 until August 2022, and Natural language processing was used to identify individual elements. Features were then displayed individually on a static dashboard developed using TableauPublic, which is available at www.equitablescience.com.In total, 130,721 papers were identified from 7,462 journals where significant underrepresentation of LMIC and Female authors was demonstrated. These findings are pervasive and show no positive correlation with the Journal’s Impact Factor.These four elements should each be seen as a new way to rank journals rather than a simplified score to be optimized. The systematic collection of these features would allow for more detailed analysis, highlight gaps in knowledge, and

Powered by Editorial Manager® and ProduXion Manager® from Aries Systems Corporation

	reflect confidence in the translation of related research. Conversion of this metric to an active pipeline would account for the fact that how we define those most at risk will change over time and quantify responses to particular initiatives. Therefore, the salient point is that continuous measurement of outcomes across groups and those investigating those outcomes will never lose importance.
Opposed Reviewers:	
Additional Information:	
Question	Response
**Financial Disclosure**<br><br>Enter a financial disclosure statement that describes the sources of funding for the work included in this submission and the role the funder(s) played. This includes grants and any commercial funding of the work or authors.<br><br>This statement will be typeset if the manuscript is accepted for publication.<br><br>*Please review the submission guidelines and the instructions link below for detailed requirements and guidance.*	The authors received no specific funding for this work.
**Competing Interests**<br><br>On behalf of all authors, disclose any competing interests that could be perceived to bias this work.<br><br>This statement will be typeset if the manuscript is accepted for publication.<br><br>*Please review the instructions link below and PLOS Global Public Health's competing interests policy to determine what information must be disclosed at submission.*	The authors have declared that no competing interests exist.
**Data Availability**<br><br>Before publication, Authors are required to make fully available and without restriction all data underlying their	All data have been sourced from open-access sources as described in the methods. These are freely available from the referenced sources without special privileges.

findings. Please see our PLOS Data Policy page for detailed information on this policy.<br><br>A **Data Availability Statement**, detailing where the data can be accessed, is required at first submission. Insert your Data Availability Statement in the box below.<br><br>Please see the data reporting section of our submission guidelines for instructions on what you need to include in your Data Availability Statement.<br><br>This statement will be typeset if the manuscript is accepted for publication.<br><br>*PLOS allows rare exemptions to address legal and ethical concerns. If you have legal or ethical restrictions, please use the box below to detail these in full sentences for the Journal team to consider.*	

Manuscript

Harvard–MIT
Health Sciences & Technology

November 25, 2022

To: PLOS Global Health

Re: A new tool for monitoring health equity; the Diversity Factor

Dear Editors,

There is increasing evidence that the *Impact Factor (IF)* has fallen foul of Goodheart's law- "When a measure becomes a target, it ceases to be a good measure". It is commonly used to evaluate journal impact through downstream citation methods. However, it is vulnerable to outliers in a given time period, thus skewing the overall calculations whilst not representing the majority of papers in the journal. In addition, there have been growing concerns regarding the strength of this index and its alignment with improving health outcomes for all populations. Digitising healthcare can transform patient care, but the current landscape does not represent those most burdened by disease. This imbalance of knowledge landscape has not been accurately quantified, nor is this regularly evaluated.

We have therefore sought to address this shortfall in this study entitled "**A new tool for monitoring health equity; the Diversity Factor**" for publication in this special edition of iScience. Our paper engages a multidisciplinary and international team of authors, including data scientists, physicians, and social scientists. This piece offers a unique perspective on the importance of diversity in health research and develops the methodology to explore this landscape.

The *Diversity Factor* covers four key areas: dataset properties, author country, author gender and departmental affiliation. Firstly, dataset characteristics heavily influence the performance of results when transferred from the original dataset. This is most evident in artificial intelligence,

Harvard–MIT
Health Sciences & Technology

yet there is evidence that it is also true for externally implemented guidelines. Secondly, we include the author's gender and their location, LMIC vs non-LMIC, as a marker of the applicability of results to these populations. Including diverse populations in the research design phase can prevent the introduction of biases that homogenous groups otherwise miss. Finally, we evaluate the authors' affiliation to analyse the collaboration between academic and industry partners. Demonstrating an apparent disconnect between these groups that mirror the poor translation of clinical devices and models.

Overall, we explore the current Diversity Factor landscape and develop a website to allow others to explore this in greater detail. Our methods are freely available for authors to expand upon this work and reproduce this research in the future. We then encapsulate this by concluding with how the Diversity factor could be implemented alongside the Impact Factor to reshape the knowledge landscape.

We thank you for your consideration and look forward to hearing from you.

Sincerely,

Leo Anthony Celi, MD, MS, MPH
Clinical Research Director, Laboratory for Computational Physiology, Harvard-MIT Division of Health Science and Technology
Associate Professor, Harvard Medical School (Part-time)
Principal Research Scientist, Massachusetts Institute of Technology
Instructor, Department of Biostatistics, Harvard T.H. Chan School of Public Health
Attending Physician, Division of Pulmonary, Critical Care, and Sleep Medicine, Beth Israel Deaconess Medical Center
Associate Program Director, Internal Medicine Residency Program, Beth Israel Deaconess Medical Center
Editor-in-Chief, *PLOS Digital Health*
lceli@mit.edu

Manuscript
Click here to access/download;Manuscript;final_diversity_factor_manuscript

A new tool for evaluating health equity in academic journals; the Diversity Factor

Authors

Jack Gallifant [1] *

Joe Zhang [2]

Stephen Whebell [3]

Justin Quion [4]

Braiam Escobar [5] #

Judy Gichoya [6] #

Karen Herrera [7] #

Ruxana Jina [8,9] #

Swathikan Chidambaram [10] #

Abha Mehndiratta [11]

Richard Kimera [12, 13] #

Alvin Marcelo [14] #

Portia Grace Fernandez-Marcelo [15] #

Juan Sebastian Osorio [16]

Cleva Villanueva [17] #

Lama Nazer [18]

Irene Dankwa-Mullan [19,20]

Leo Anthony Celi [21, 22, 23]

Affiliations

[1] Department of Intensive Care, Imperial College London NHS Trust, London, UK

[2] Institute of Global Health Innovation, Imperial College London, London, UK

[3] Intensive Care Unit, Townsville University Hospital, Townsville, QLD, Australia

[4] University of the East Ramon Magsaysay Memorial Medical Center, Philippines.

[5] Escuela de Ingeniería de Antioquia, Envigado, Colombia

[6] School of Medicine, Emory University, 1364 Clifton Road NE, 30322, Atlanta, USA

[7] Military Hospital - Nicaragua, Head of the Quality Department, Teacher and researcher Faculty of Medicine

[8] The Epidemiology and Surveillance Section, National Institute for Occupational Health, National Health Laboratory Services, Gauteng Region, South Africa

[9] The Wits School of Public Health, Faculty of Health Sciences, University of the Witwatersrand, Johannesburg, South Africa

[10] Department of Surgery and Cancer, Imperial College London, London, UK.

[11] Center for Global Development, Washington DC, USA.

[12] Department of Information Technology, Faculty of Computing and Informatics, Mbarara University of Science and Technology, Uganda.

[13] Department of Advanced Convergence, Handong Global University, South Korea.

[14] University of the Philippines Manila, Philippines

[15] Department of Family and Community Medicine, College of Medicine, University of the Philippines Manila, Philippines

[16] ScienteLab, Bogota, Colombia.

[17] Instituto Politecnico Nacional. Escuela Superior de Medicina. Mexico City. Mexico

[18] King Hussein Cancer Center, Amman, Jordan

[19] Merative, & Center for AI, Research, and Evaluation, IBM Watson Health, Cambridge, Massachusetts, USA.

[20] Department of Health Policy and Management, Milken Institute School of Public Health, George Washington University, Washington DC, USA.

[21] Massachusetts Institute of Technology, Laboratory for Computational Physiology, Cambridge, MA.

[22] Beth Israel Deaconess Medical Center, Division of Pulmonary, Critical Care, and Sleep Medicine, Boston, MA.

[23] Department of Biostatistics, Harvard T.H. Chan School of Public Health, Boston, MA.

# Authors contributed equally to the manuscript.

* Corresponding author

Correspondence: jack.gallifant@nhs.net

Abstract

Current methods to evaluate a journal's impact rely on the downstream citation mapping used to generate the Impact Factor. This approach is a fragile metric prone to being skewed by outlier values and does not speak to a researcher's contribution to furthering health outcomes for all populations. Therefore, we propose the implementation of a Diversity Factor to fulfill this need and supplement the current metrics. It is composed of four key elements: dataset properties, author country, author gender and departmental affiliation. Due to the significance of each individual element, they should be assessed independently of each other as opposed to being combined into a simplified score to be optimized. Herein, we discuss the necessity of such metrics, provide a framework to build upon, evaluate the current landscape through the lens of each key element and publish the findings on a freely available website that enables further evaluation.

The OpenAlex database was used to extract the metadata of all papers published from 2000 until August 2022, and Natural language processing was used to identify individual elements. Features were then displayed individually on a static dashboard developed using TableauPublic, which is available at www.equitablescience.com.

In total, 130,721 papers were identified from 7,462 journals where significant underrepresentation of LMIC and Female authors was demonstrated. These findings are pervasive and show no positive correlation with the Journal's Impact Factor.

The systematic collection of the Diversity Factor concept would allow for more detailed analysis, highlight gaps in knowledge, and reflect confidence in the translation of related research. Conversion of this metric to an active pipeline would account for the fact that how we define those most at risk will change over time and quantify responses to particular initiatives. Therefore, continuous measurement of outcomes across groups and those investigating those outcomes will never lose importance. Moving forward, we encourage further revision and improvement by diverse author groups in order to better refine this concept.

Background

The last decade has seen our capacity to store, analyse and distribute health data grow exponentially, especially with the growing use of artificial intelligence (AI), yet healthcare has tried and failed to implement it in a successful manner. The current AI landscape is ever-expanding and many of the current models are either still in the prototype stage [1,2] or exhibit substandard performance, particularly in the cases of sepsis and COVID-19 [3,4]. More worryingly, AI has inherent bias, introduced by both the data and those who created it, and it is no surprise that it can disproportionately affect minorities [5,6]. This has led to the call for greater transparency in the model development phase, improved data sharing, and more diversity among research groups to safeguard against such biases [7]. However, these changes have not yet reached the journal-level as the current metrics used to evaluate research and journal impact do not focus on such factors. Furthermore, the ability to provide a complete measure of health research's significance, penetrance, and relevance has been debated for decades [8].

Initially designed to track citations of articles by authors and journals, the impact factor (IF) used to judge the importance of scientific or academic publications and the journal itself. Though the IF accounts for variations in publishing volumes between journals, the impact on the population or a community had little relevance to the indices [9–12]. Currently, the IF has transformed into a proxy for the quality of individual articles even though highly cited papers skew calculations. As a result, journal figures do not represent the majority of papers in a specific journal [13–16]. In addition, the IF has several limitations, such as not accounting for the citation density of fields or fluctuations in publication practices, for example, during the COVID-19 pandemic, which inflated critical care journals' impact factors [17,18]. Moreover, leading academic journals have gamed the system to improve their own IF through self-citation, a practice which is equally common among leading authors [19,20].

Despite the emphasis on IF, and citations as proxy, they do not equate to scientific excellence or the advancement of health research that improves outcomes for all [21]. There is increasing evidence of disparities in outcomes across demographic groups, where the COVID-19 pandemic was a prime example [22,23]. These disparities are further reinforced by nonrepresentative research, in regards to the lack of diversity in both researchers and questions [24]. Having a research group that better represents their population in question allows for better coverage of multiple problem-solving styles and a better understanding of the problems they face [25,26]. Furthermore, increased ethnic diversity has been found to be strongly correlated with scientific impact and can help lead to better science [27].

Yet, despite the heavy emphasis on the importance of diversity, there is no objective measure for tracking progress towards inclusivity in science or for evaluating who contributes to health research [7,28]. A shift from a single citation-based metric to using several different metrics that provide a more complete perspective on factors aligned with scientific excellence based on contribution to advancing inclusivity and diversity, improving health outcomes, and achieving equity is therefore necessary.

Characteristics of a Diversity Factor for assessing Journal Impact

This proposed Diversity Factor (DF) should provide an alternative means of tracking accurate and reliable contributions to health research aligned with the impact on the global community or population in addition to offering an approach to facilitate scientific excellence that is unbiased, representative, and impactful. Important factors that should be considered in evaluating the literature include features related to Publications, Authorship, and Research Oversight. These are explored in Table 1 along with guiding questions that describe the feature's respective characteristics. It is important to note that this is merely a proposal and should serve as a foundation to build upon. Additionally, this proposed Diversity Factor should

serve as a supplement to the currently used metrics and not as a replacement. Scoring of this concept is also left for future revisions. For this paper, each key element is reviewed independent of the others.

In this paper, the authors focused on developing tools that allow for the evaluation of the current landscape in regard to four key elements: dataset properties, author country (LMIC status), author gender (M/F) and organisational affiliation (including multidisciplinary team approach).

1. Dataset Characteristics

Data selection is an inevitable component of research; not all data can be captured and instead strict selection is necessary to answer the research question at hand. However, this inherently creates a restricted view that affects the conclusions drawn, irrespective of domain. The characteristics of datasets used to inform clinical decisions are vital as these conclusions will likely have the greatest relevance to the populations studied. This can be further exacerbated as AI has the potential to perpetuate these biases if left unchecked [29]. The authors propose that datasets used in health research should be mapped to highlight geographic and academic areas of data poverty, expose underlying knowledge gaps, and draw attention to imbalanced datasets [30]. These include gender imbalance, race-ethnicity, language preference, age, and geography. The current impact factor rewards citations equally but, determining who has read, utilized, and has been ‘impacted’ by the research is not as simple as implied. Most data being analyzed to guide healthcare is derived from a few centers, almost exclusively based in High-Income Countries [31]. As such, increasing the global impact of research is vital, and the increasing adoption of technology has created the potential to democratize health research. More effort should then be placed on increasing the diversification of the data pool used to design clinical guidelines and develop tools that provide beneficial outcomes for all, and not just a select few countries.

2. Author Country

Author’s previous experiences, surrounding culture, and the associated team will significantly shape projects. Thus, when considering how to evaluate an author group, it is important to heavily weigh those with increased diversity, as these groups will better reflect the populations in question as well as having a wider range of problem-solving abilities and perspectives. Evaluating the spread in the country of affiliations within studies can speak to the cognitive diversity of the teams and the likelihood of methodology and results transferring to that area. The authors designed the study methodology, conducted the analysis, interpreted the findings and presented these in an organized manner. Throughout these stages, biases can be introduced by influencing selection strategies, modes of analyses and presentation of results. Authors working in one country who analyze datasets from another have inherent limitations due to an incomplete understanding of the context and culture of the studied subjects. Including diverse perspectives can maximize the scope for identifying potential biases and ensuring the results produced are applicable to multiple locations. Notably, historical racial prejudices have resulted in disparities in clinical outcomes between demographics and including a variety of ethnicities would improve safeguarding against introducing similar biases [32]. It is commonplace for knowledge to centralize, with intellectual centers producing multitudes of research. These ‘Ivory Towers' often overrepresent a particular demographic that is inconsistent with the experience or backgrounds of those most burdened by disease. Increasing diversity within author groups, especially within institutions, can help combat this resulting homogeneity of thought.

3. Author Gender

Diversity is more than just increasing the number of ethnicities within the author group. Traditionally, academia has been a male-dominated field, well-documented across multiple fields [33,34]. However within recent years, this trend has been shifting - more and more women are joining the field. However, gender-parity has not yet been reached and given the current trends will take several more years unless there are active, intentional changes. In this

study, an algorithm trained to identify ‘gender’ as Male or Female was utilized. However, as further research is done to refine this proposal, expanding this definition to include other genders and the distinction between sex and gender would be ideal.

4. Organizational or Departmental affiliation

Most health research has traditionally been conducted in a few institutions with the necessary funding and access to data. Considering the centralization of knowledge and the overrepresentation of certain demographics found in these ivory towers, there has been a recent shift to increase engagement with local stakeholders and increase patient public involvement. Further, healthcare is increasingly becoming a multi-disciplinary field as a result of the recognition that socioeconomic factors play significant roles in health outcomes. As such, multidisciplinary teams play an important role in bridging professional boundaries and breaking down the barriers of competing cultural and organizational differences will improve the translational gap in medical devices and root academic work in deployable applications. There are currently divides between clinical, academic, and commercial research that often leaves everyone feeling that data is out of reach. Bridging the gap between commercial and non-commercial fields will improve the translational gap in medical devices and root academic work in deployable applications. Understanding the current breakdown of institutional affiliation, whether it is academic or commercial, is necessary to see if academia is responding to this multidisciplinary call. Expansion of this definition to include the composition of expertise will also be vital, for example, the interaction of machine learning engineers and social scientists in the field of AI.

Analysis of Diversity Factor in Journals with Global Reach

To evaluate the current diversity factor landscape, we used the OpenAlex database consisting of the metadata of all papers published from 2000 until August 2022 [35]. The entire database

was downloaded locally, where metadata, including author name, affiliation, and study abstract, was extracted. Only journals identified by SCImago were included in the analysis.

Dataset Characteristics were not widely named in study abstracts, and due to the lack of freely available full papers, we could not identify this factor reproducibly. The code to implement this feature evaluation has been validated and is freely available for future use [36]. However, due to these limitations, it has been excluded from the analysis portion of this study.

Author affiliations were identified and geocoded using OpenAlex identification of more than 100,000 research producers in the Research Organisation Registry [37]. We geocoded raw affiliation strings for affiliations with no match using a custom Nominatim API [38]. Geographic locations were matched in 88% of author instances. Thereafter, Author Countries were grouped by income status according to the World Bank; Low-Middle Income Country (LMIC) or non-LMIC Country [39].

Enriched metadata is produced using fine-tuned Natural Language Processing models (BERT-Pubmed) for research classification and entity extraction, as described elsewhere [1]. Affiliation strings derived from this process were then parsed for information on departmental affiliation that were then categorized into commercial and academic organizations.

We identified the author's gender using several APIs that demonstrate state-of-the-art performance in validation studies on non-English names, including Gender-API and Genderize [40,41]. Gender matching was conducted using the first name and affiliation country, with 84% of entries matched. The female: male author ratio was calculated for each paper, and then a mean was calculated for each journal and time period.

Features are displayed individually, and a hypothesis of aggregation is discussed below. Descriptive statistics for each of the three included features is displayed in a subset of eight journals that cover the broadest ranges of speciality, Impact Factor, and traditional prestige.

A static dashboard using over 7539 journals was then developed using TableauPublic to represent the full diversity factor landscape and to allow for effect modifiers (open-access and funding sources) to be evaluated; this is available at www.equitablescience.com.

Results

Since the year 2000, 130,721 papers have been identified from 7539 journals, a majority of which are from authors based in North America or Central Asia and Europe. In 2021, there were 0.68 and 0.73 authors per paper per region, respectively, compared to under 0.1 in each of Latin America, the Caribbean, South Asia, and Africa. Underrepresentation of female authors is seen throughout. In 2000, median female:male author ratio was 0.40 and has increased over time to 0.78 in 2021. There are a few countries where journals have reached gender parity, such as Portugal (1.13) and Cuba (1.09). However, despite this trend towards greater representation for female authors, it is unclear how many journals, if any, will reach gender-parity in the next five years given the rate of improvement. Those most at risk of being 'left behind' are primarily from low-income countries though some countries such as Japan (0.24) and South Korea (0.26) face such obstacles as well.

Authors from low-middle-income countries are sorely underrepresented as well. In 2021, there were over 5 million authors from high-income countries. In comparison, upper-middle-income countries had 1.5 million authors, lower-middle-income countries had 467,323, and low-income countries had 27,080 authors. Across all journals, the LMIC:non-LMIC author ratio was 0.014 in 2021, the result of a slow improvement since 2000 (Figure 1B)

Interestingly, these trends of female author and LMIC author underrepresentation are reversed when looking at a subset of open-access journals. When comparing the top 25 most-impactful open access journals and non-open access journals, open access journals

consistently had higher proportions of female authors and authors from LMICs over the past two decades.

Table 2 summarizes the findings from a selection of well-known journals from 2021. The female:male ratio of authors in this subset mirrors the consistent pattern of female author underrepresentation seen in all journals, with none from this subset averaging over 1. Author representation from LMICs is consistently low as well, with most journals under 1:10. In addition, most authors were from academic or non-commercial organizations, highlighting the lack of multidisciplinary collaboration.

Discussion

In this paper, the concept of a Diversity Factor was proposed as a supplemental metric of measuring a journal's contribution to the research landscape, focusing on diversity, equity, inclusion, and impact on the studied community or population. Analysis of the data under the lens of the key elements of a proposed Diversity Factor reveal unsurprising results. Female authors and authors from LMICs are sorely underrepresented. While trends are improving for both demographics, several obstacles still stand in the way. Academia is becoming more and more centralized especially in high-income countries. As it does so, it becomes more difficult to penetrate, especially when considering the trends of self-citation.

What steps to take in order to help improve inclusivity and diversity lie outside the scope of this paper. However, the reversal of trends seen when comparing open-access journals to non-open-access journals may be an angle worth further investigation. These results are most likely due to the easier accessibility found with the open-access model but are worth noting.

The next steps for implementing a diversity factor would include the availability of dataset characteristics, detailed funding sources, patents and downstream policy impact, and citation mapping. This would allow for a better understanding of who is impacted and who is causing the impact. Additionally, refining the use of author country to a place of birth or time spent in a country would account for those in LMICs who emigrate to other institutions, which is not uncommon. Further, the improvement of gender data or NLP algorithms to account for author gender compared to sex would be another step forward. The operationalisation of this tool would rely on interest from journals and researchers in collaborating to permit this data to be published on each journal's website and centrally for evaluation. The heterogeneity in the findings between these four diversity metrics and between journals likely means that these four values should not be combined into one ‘diversity value’. Instead, they should be evaluated and compared individually as seen in Figure 2, or other visual tools such as in a star diagram.

Additionally, we propose having such values rechecked yearly to offer checkpoints and track progress as new research is published and populations evolve. Routine reporting would further allow for more detailed analysis to be performed, highlight gaps in knowledge, and reflect confidence in the translation of related research. This is particularly true at the health policy level, where it is known that the social determinants of health vary greatly between countries; therefore, clinical decisions and public health decisions should be made based on information more representative of these populations. It is important to acknowledge that there is not one type of bias, nor one group affected by bias, but many types of bias and many groups that can be biased against. Diversity is not a box-ticking exercise but an essential safeguard against potential biases, especially in countries with greater ethnicity, cultural diversity and particular socio-demographic characteristics. Promotion of these features would encourage thoughtful discourse on how study designs and data characteristics can affect different groups so that researchers and organizations can build the right team for the specific project and its risks. Furthermore,

integration of the Diversity Factor can encourage collaboration with LMICs that could reshape the knowledge landscape through the dissemination of work partnered with LMICs.

Our goal was to evaluate the current diversity factor landscape to the highest degree permissible by the availability of data and the current standards of metadata. Data set characteristics were not widely available, which did not permit the evaluation of this important feature. However, we have provided the tools to implement this feature if made available in the future. Gender was determined using NLP, which has demonstrated good performance across countries; however, the ground truth is not available for these studies and so cannot be confirmed. SCImago Journal Ranking is used in place of traditional impact factor due to the open source nature and availability of the feature, there is a similarity between the two, but we also acknowledge differences. We recognise these tools are imperfect, but we hope they provide a 'big picture' view of the global research landscape and demonstrate what is possible in the future with greater open access to journals and why these metrics could greatly promote the drive for equitable science.

The four elements used in this paper uncovers a bleak reality unseen by citation-based metrics. Academia as it is now, and the healthcare systems that it shapes, cannot equitably and justly provide for all if it is not reflective of the populations in question. And in order to do that, it is necessary to change the way science evaluates its efforts. The current methods of measuring journal impact are far from ideal and fail to provide an estimate of author and dataset diversity. While diversity should not be the only consideration of researchers or journals, it should complement the downstream citation impact. Yet, tracking who participates in the conversations that shape healthcare and where our opinions are being formed should be monitored and evaluated transparently and publicly.

The Diversity Factor is a call to action for improved representation and the encouragement of diverse perspectives in health research to prevent the perpetuation of biases against

subgroups and the advancement of scientific excellence that works for all. It reminds journals and authors to assess how thoughts and data reach the manuscript and whether they consider all perspectives, not just those available at hand. Otherwise, we will continue learning and practising medicine in an echo chamber created by the few Ivory Tower Academics with access to the resources and data required to advance the field left in the hands of a select few institutions.

These findings and a more detailed analysis from this paper are available online to permit comparisons across open access, funding sources and specific use cases such as COVID-19 [42]. Moreover, the code is also freely available online in the interest of reproducibility, the addition of other features, and for later conversion to an active pipeline [36].

Acknowledgements

We would like to acknowledge the contribution of the OpenAlex database, without which this project would not have been possible.

Author Contributions

Conceptualization, JG, JZ, and LC.; Methodology and Formal Analysis, JG, JZ, SW and LC; Writing – Original Draft, JG and JZ; Writing – Review & Editing, All authors.

Declaration of interests

The authors declare no conflicts of interest.

Figure and scheme legends

Figure 1. The mean value for **A)** median Female: Male author ratio on a paper per journal each year from 2000-2021, **B)** median LMIC: non-LMIC author ratio per journal each year from 2000-2021.

Figure 2. Comparison of the individual features of the Diversity factor for two journals, from https://equitablescience.com/dashboards/diversity/.

Tables

Table 1. Elements for Assessing Journal Contribution to Scientific Excellence in Diversity, Equity and Inclusion.

**Category**	**Element**	**Guiding Questions**
**Publications**	Diversity	Do studies explore health, determinants of health and underlying factors driving improved outcomes for diverse population groups?
	Equity	Do publications showcase research relevant to under-represented communities and populations?
	Inclusion	Have publications promoted inclusive and multidisciplinary research methods?
	Datasets	What are the definitions and distributions of age, race, ethnicity, gender, language, and geography, included in the study participants?
**Authorship**	Author identity, country or origin	Is there a wide range of author cultures, experience and expertise, including country or origin and low-middle income countries (LMICs)?
	Author Gender	Is there a balanced gender distribution on average among authors, and first/last authors?

	Author Organization and Affiliation	Is there a diverse range of organizations, including minority serving institutions, industry and academia?
	Community experts	Has the published research been conducted with community experts, and are they named co-authors?
**Research Oversight**	Journal Editors and Reviewers	Has the journal developed an accountability system to measure and ensure diversity among editors and reviewers?
	Review Process	Does a journal have a system to identify and respond to potential bias in the review process?
	Communication and Dissemination	Are the results of the above questions easily accessible and transparent for researchers and the general public to review?

Table 2. Comparison of median diversity factors across journals in 2021

Journal	SJR (IF)	Female: Male *	LMIC: non-LMIC author *	Commercial: Non-commercial affil.*
BMJ	2291 (93)	0.43	0.03	0.02
JAMA	6076 (157)	0.40	0.01	0.03
Lancet	6024 (202)	0.37	0.15	0.03
Nature Medicine	15652 (87)	0.38	0.02	0.05
NEJM	24161 (176)	0.36	0.03	0.09
PLoS Medicine	24907 (12)	0.42	0.12	0.01
npj Digital Medicine	4180 (15)	0.30	0.02	0.12
The Lancet. Digital health	3326 (37)	0.31	0.03	0.05

SJR= Scimago Journal Ranking, Average number of weighted citations received in a year by articles published in a journal in the year 2021. IF = Impact Factor. * Ratio of mean values per paper for each journal 2dp. Commercial affil.= the mean number of authors per paper from a commercial company.

References

1. Zhang J, Whebell S, Gallifant J, Budhdeo S, Mattie H, Lertvittayakumjorn P, et al. An interactive dashboard to track themes, development maturity, and global equity in clinical artificial intelligence research. The Lancet Digital Health. 2022;4: e212–e213. doi:10.1016/S2589-7500(22)00032-2

2. van de Sande D, van Genderen ME, Huiskens J, Gommers D, van Bommel J. Moving from bytes to bedside: a systematic review on the use of artificial intelligence in the intensive care unit. Intensive Care Med. 2021;47: 750–760. doi:10.1007/s00134-021-06446-7

3. Wong A-KI, Charpignon M, Kim H, Josef C, de Hond AAH, Fojas JJ, et al. Analysis of Discrepancies Between Pulse Oximetry and Arterial Oxygen Saturation Measurements by Race and Ethnicity and Association With Organ Dysfunction and Mortality. JAMA Netw Open. 2021;4: e2131674–e2131674. doi:10.1001/jamanetworkopen.2021.31674

4. Singh K, Valley TS, Tang S, Li BY, Kamran F, Sjoding MW, et al. Evaluating a Widely Implemented Proprietary Deterioration Index Model among Hospitalized Patients with COVID-19. Ann Am Thorac Soc. 2021;18: 1129–1137. doi:10.1513/AnnalsATS.202006-698OC

5. HealthLeaders. Root of some health disparities may be buried in technology. [cited 30 Nov 2022]. Available: https://www.healthleadersmedia.com/technology/root-some-health-disparities-may-be-buried-technology

6. Gianfrancesco MA, Tamang S, Yazdany J, Schmajuk G. Potential Biases in Machine Learning Algorithms Using Electronic Health Record Data. JAMA Intern Med. 2018;178: 1544–1547. doi:10.1001/jamainternmed.2018.3763

7. Oh SS, Galanter J, Thakur N, Pino-Yanes M, Barcelo NE, White MJ, et al. Diversity in Clinical and Biomedical Research: A Promise Yet to Be Fulfilled. PLoS Med. 2015;12: e1001918. doi:10.1371/journal.pmed.1001918

8. Singh Chawla D. What's wrong with the journal impact factor in 5 graphs. nature index; 2018. Available: https://www.nature.com/nature-index/news-blog/whats-wrong-with-the-jif-in-five-graphs

9. Garfield E. Citation Analysis as a Tool in Journal Evaluation. Science. 1972;178: 471–479. doi:10.1126/science.178.4060.471

10. Garfield E, Sher I. Genetics Citation Index. Annu Rep Can Inst Sci Tech Inf/Rapp Annu Inst Can Inf Sci Tech. 1963. Available: http://www.garfield.library.upenn.edu/essays/v7p515y1984.pdf

11. Brodman E. Choosing Physiology Journals. Bull Med Libr Assoc. 1944;32: 479–483.

12. Garfield E. Citation Analysis as a Tool in Journal Evaluation. Science. 1972;178: 471–479. doi:10.1126/science.178.4060.471

13. Bornmann L, Marx W. How good is research really? EMBO Rep. 2013;14: 226–230. doi:10.1038/embor.2013.9

14. Paulus FM, Cruz N, Krach S. The Impact Factor Fallacy. Front Psychol. 2018;9. doi:10.3389/fpsyg.2018.01487

15. Bornmann L, Marx W, Gasparyan AY, Kitas GD. Diversity, value and limitations of the journal impact factor and alternative metrics. Rheumatol Int. 2012;32: 1861–1867. doi:10.1007/s00296-011-2276-1

16. Dimitrov JD, Kaveri SV, Bayry J. Metrics: journal's impact factor skewed by a single paper. Nature. 2010;466: 179–179. doi:10.1038/466179b

17. Di Girolamo N, Meursinge Reynders R. Characteristics of scientific articles on COVID-19 published during the initial 3 months of the pandemic. Scientometrics. 2020;125: 795–812. doi:10.1007/s11192-020-03632-0

18. Fraser N, Brierley L, Dey G, Polka JK, Pálfy M, Nanni F, et al. The evolving role of preprints in the dissemination of COVID-19 research and their impact on the science communication landscape. PLoS Biol. 2021;19: e3000959. doi:10.1371/journal.pbio.3000959

19. Mishra S, Fegley BD, Diesner J, Torvik VI. Self-citation is the hallmark of productive authors, of any gender. PLoS One. 2018;13: e0195773. doi:10.1371/journal.pone.0195773

20. Taşkın Z, Doğan G, Kulczycki E, Zuccala AA. Self-Citation Patterns of Journals Indexed in the Journal Citation Reports. J Informetr. 2021;15: 101221. doi:10.1016/j.joi.2021.101221

21. Ray KS, Zurn P, Dworkin JD, Bassett DS, Resnik DB. Citation bias, diversity, and ethics. Account Res. 2022; 1–15. doi:10.1080/08989621.2022.2111257

22. Sze S, Pan D, Nevill CR, Gray LJ, Martin CA, Nazareth J, et al. Ethnicity and clinical outcomes in COVID-19: A systematic review and meta-analysis. eClinicalMedicine. 2020;29. doi:10.1016/j.eclinm.2020.100630

23. Magesh S, John D, Li WT, Li Y, Mattingly-app A, Jain S, et al. Disparities in COVID-19 Outcomes by Race, Ethnicity, and Socioeconomic Status: A Systematic Review and Meta-analysis. JAMA Network Open. 2021;4: e2134147–e2134147. doi:10.1001/jamanetworkopen.2021.34147

24. Seastedt KP, Schwab P, O'Brien Z, Wakida E, Herrera K, Marcelo PGF, et al. Global healthcare fairness: We should be sharing more, not less, data. PLOS Digital Health. 2022;1: e0000102. doi:10.1371/journal.pdig.0000102

25. Aminpour P, Schwermer H, Gray S. Do social identity and cognitive diversity correlate in environmental stakeholders? A novel approach to measuring cognitive distance within and between groups. PLOS ONE. 2021;16: e0244907. doi:10.1371/journal.pone.0244907

26. Meissner P, Wulf T. The effect of cognitive diversity on the illusion of control bias in strategic decisions: An experimental investigation. European Management Journal. 2017;35: 430–439. doi:10.1016/j.emj.2016.12.004

27. AlShebli BK, Rahwan T, Woon WL. The preeminence of ethnic diversity in scientific collaboration. Nat Commun. 2018;9: 5163. doi:10.1038/s41467-018-07634-8

28. Striving for Diversity in Research Studies. N Engl J Med. 2021;385: 1429–1430. doi:10.1056/NEJMe2114651

29. Panch T, Mattie H, Atun R. Artificial intelligence and algorithmic bias: implications for health systems. J Glob Health. 2019;9: 010318. doi:10.7189/jogh.09.020318

30. Ibrahim H, Liu X, Zariffa N, Morris AD, Denniston AK. Health data poverty: an assailable barrier to equitable digital health care. The Lancet Digital Health. 2021;3: e260–e265. doi:10.1016/S2589-7500(20)30317-4

31. Smyth B, Trongtrakul K, Haber A, Talbot B, Hawley C, Perkovic V, et al. Inequities in the global representation of sites participating in large, multicentre dialysis trials: a systematic review. BMJ Global Health. 2019;4: e001940. doi:10.1136/bmjgh-2019-001940

32. Cerdeña JP, Plaisime MV, Tsai J. From race-based to race-conscious medicine: how anti-racist uprisings call us to act. Lancet. 2020;396: 1125–1128. doi:10.1016/S0140-6736(20)32076-6

33. Vincent J-L, Juffermans NP, Burns KEA, Ranieri VM, Pourzitaki C, Rubulotta F. Addressing gender imbalance in intensive care. Critical Care. 2021;25: 147. doi:10.1186/s13054-021-03569-7

34. The Lack of Diversity in Healthcare on JSTOR. [cited 14 May 2023]. Available: https://www.jstor.org/stable/26894210

35. Priem J, Piwowar H, Orr R. OpenAlex: A fully-open index of scholarly works, authors, venues, institutions, and concepts. 2022; arXiv:2205.01833.

36. whizzlab. health_ai_question-answer. 2022 [cited 1 Sep 2022]. Available: https://github.com/whizzlab/health_ai_question_answer

37. Research Organization Registry. 2022 [cited 1 Sep 2022]. Available: https://ror.org/

38. Nominatim team. Nominatim. 2022 [cited 1 Sep 2022]. Available: https://nominatim.org/

39. The World Bank. World Bank Open Data,. 2022 [cited 1 Sep 2022]. Available: https://data.worldbank.org/

40. Gender API Team. Gender API. 2022 [cited 1 Sep 2022]. Available: https://gender-api.com/

41. Genderize Team. Genderize API. 2022. Available: https://genderize.io/

42. Equitable Science. 2022 [cited 1 Nov 2022]. Available: https://equitablescience.com/

Figure 2

Click here to access/download;Figure;Fig_2.tiff

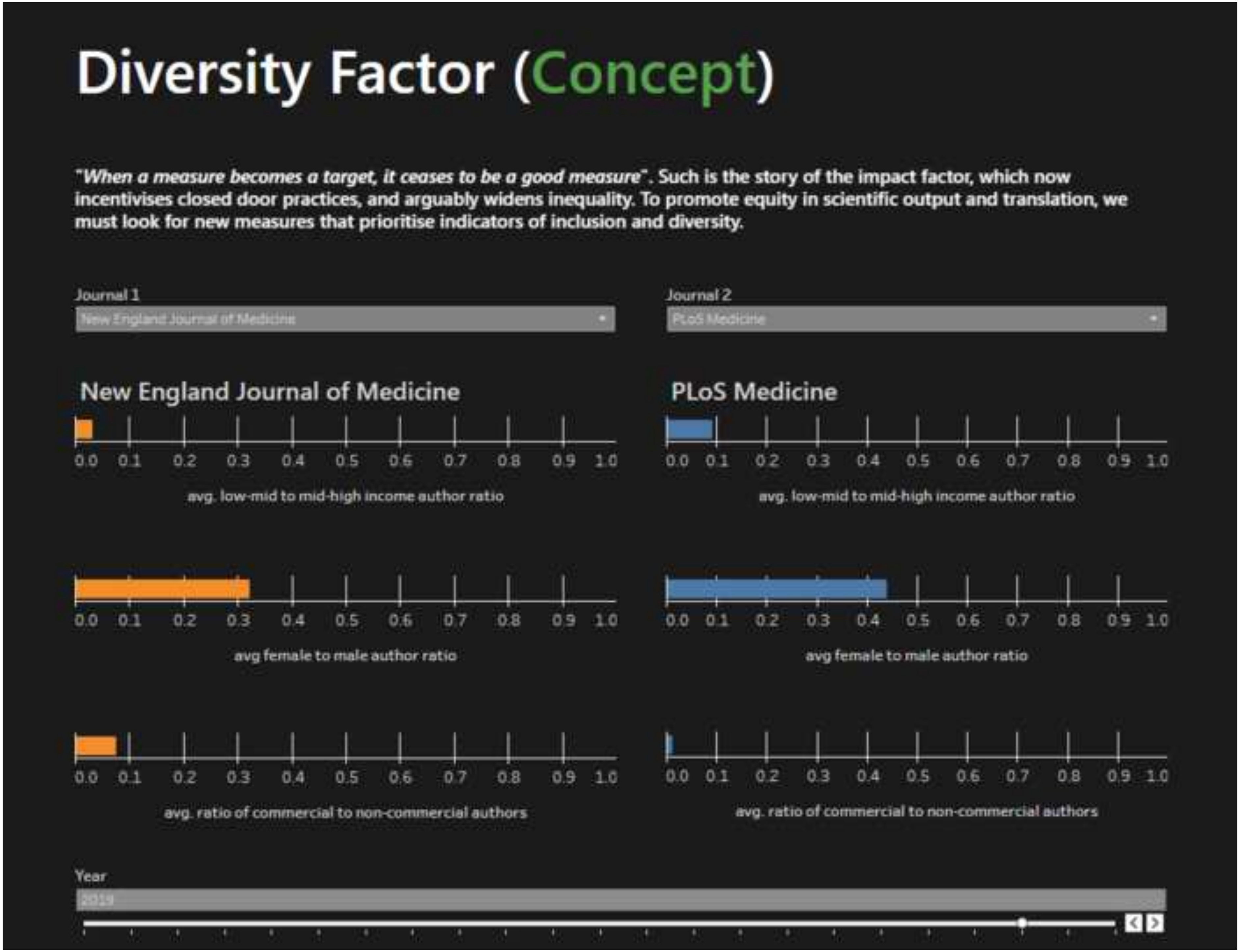

Figure 1

Click here to access/download;Figure;Fig_1.tiff.tif

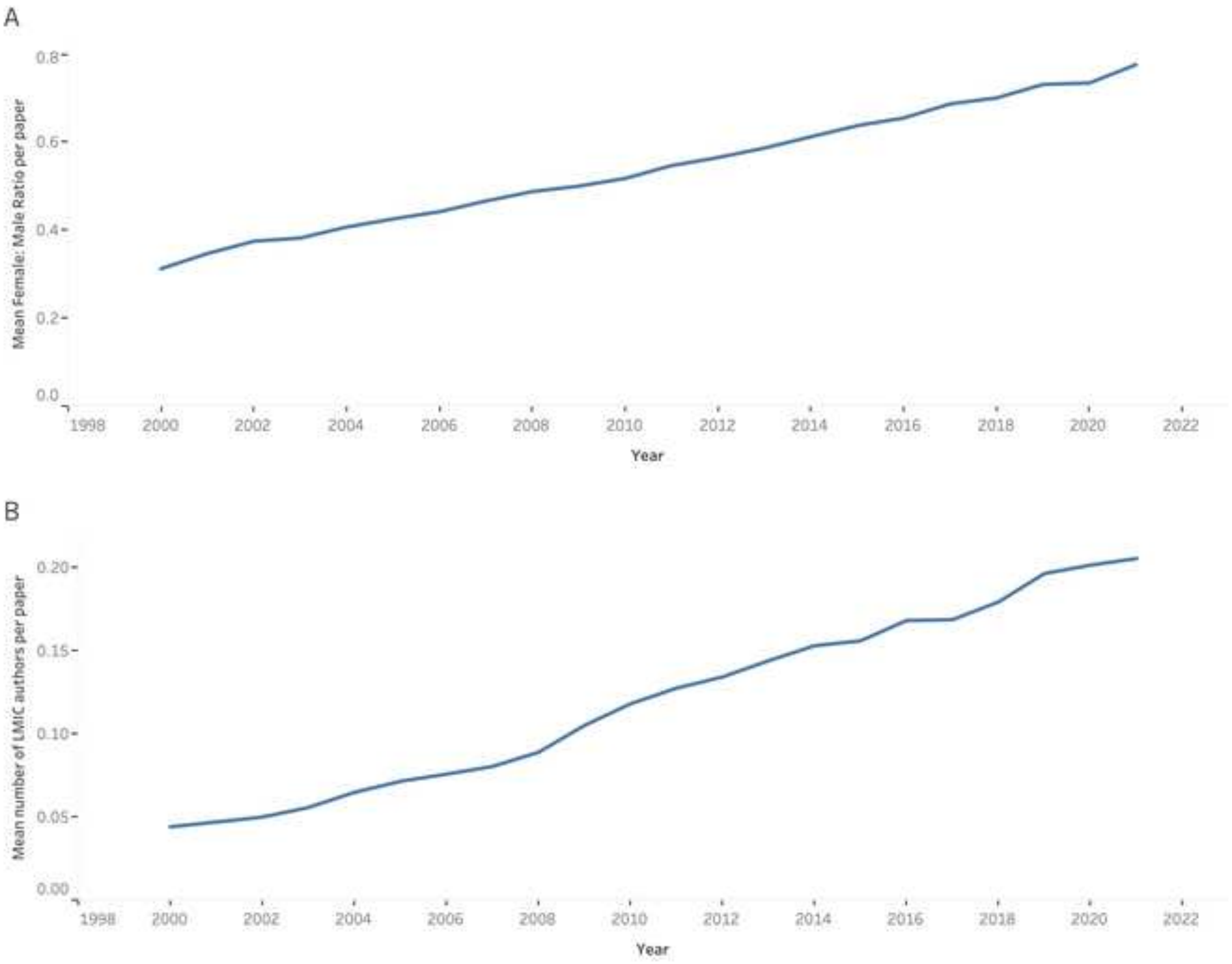

tracked manuscript

A new tool for evaluating health equity in academic journals; the Diversity Factor

Authors

Jack Gallifant [1 *]

Joe Zhang [2]

Stephen Whebell [3]

Justin Quion [4]

Braiam Escobar [54 #]

Judy Gichoya [65 #]

Karen Herrera [67 #]

Ruxana Jina [7, 8,98 #]

Swathikan Chidambaram [109 #]

Abha Mehndiratta [1122]

Richard Kimera [120, 131 #]

Alvin Marcelo [134 #]

Portia Grace Fernandez-Marcelo [154 #]

Juan Sebastian Osorio [1623]

Cleva Villanueva [157 #]

Lama Nazer [186]

Irene Dankwa-Mullan [197,2018]

Leo Anthony Celi [2119,, 202, 213]

Formatted: Superscript

Formatted: Not Highlight

Affiliations

[1] Department of Intensive Care, Imperial College London NHS Trust, London, UK

[2] Institute of Global Health Innovation, Imperial College London, London, UK

[3] Intensive Care Unit, Townsville University Hospital, Townsville, QLD, Australia

[4] University of the East Ramon Magsaysay Memorial Medical Center, Philippines.

[5] Escuela de Ingeniería de Antioquia, Envigado, Colombia

[6~~5~~] School of Medicine, Emory University, 1364 Clifton Road NE, 30322, Atlanta, USA

[7~~6~~] Military Hospital - Nicaragua, Head of the Quality Department, Teacher and researcher Faculty of Medicine

[8~~7~~] The Epidemiology and Surveillance Section, National Institute for Occupational Health, National Health Laboratory Services, Gauteng Region, South Africa

[9~~8~~] The Wits School of Public Health, Faculty of Health Sciences, University of the Witwatersrand, Johannesburg, South Africa

[10~~9~~] Department of Surgery and Cancer, Imperial College London, London, UK.

[11] Center for Global Development, Washington DC, USA.

[12~~0~~] Department of Information Technology, Faculty of Computing and Informatics, Mbarara University of Science and Technology, Uganda.

[13~~1~~] Department of Advanced Convergence, Handong Global University, South Korea.

~~[12] Department of Radiology, Stanford University, 300 Pasteur Drive, Stanford, CA, 94305-5281, USA.~~

[14~~3~~] University of the Philippines Manila, Philippines

[15~~4~~] Department of Family and Community Medicine, College of Medicine, University of the Philippines Manila, Philippines

[16] ScienteLab, Bogota, Colombia.

[17~~5~~] Instituto Politecnico Nacional. Escuela Superior de Medicina. Mexico City. Mexico

[18~~6~~] King Hussein Cancer Center, Amman, Jordan

Formatted: Not Highlight

[19] Merative, & Center for AI, Research, and Evaluation, IBM Watson Health, Cambridge, Massachusetts, USA.

[20] Department of Health Policy and Management, Milken Institute School of Public Health, George Washington University, Washington DC, USA.

[21] Massachusetts Institute of Technology, Laboratory for Computational Physiology, Cambridge, MA.

[22] Beth Israel Deaconess Medical Center, Division of Pulmonary, Critical Care, and Sleep Medicine, Boston, MA.

[23] Department of Biostatistics, Harvard T.H. Chan School of Public Health, Boston, MA.

~~[22] Center for Global Development, Washington DC, USA.~~

~~[23] ScienteLab, Bogota, Colombia.~~

# Authors contributed equally to the manuscript.

***** Corresponding author

Correspondence: jack.gallifant@nhs.net

Abstract

Current methods to evaluate a journal's impact rely on the downstream citation mapping used to generate the Impact Factor. This approach is a fragile metric prone to being skewed by outlier values and does not speak to a researcher's contribution to furthering health outcomes for all populations. Therefore, we propose the implementation of a Diversity Factor to fulfill this need and supplement the current metrics. It is composed of four key elements: dataset properties, author country, author gender and departmental affiliation. Due to the significance of each individual element, they should be assessed independently of each other as opposed to being combined into a simplified score to be optimized. Herein, we discuss the necessity of such metrics, provide a framework to build upon, evaluate the current landscape through the lens of each key element ~~Therefore, we developed the Diversity Factor to fulfil this need and comprised four key elements: dataset properties, author country, author gender and departmental affiliation. Herein, we develop the methodology to reproducibly calculate these elements, evaluate the current landscape~~ and publish the findings on a freely available website that enables further evaluation.

The OpenAlex database was used to extract the metadata of all papers published from 2000 until August 2022, and Natural language processing was used to identify individual elements. Features were then displayed individually on a static dashboard developed using TableauPublic, which is available at www.equitablescience.com.

In total, 130,721 papers were identified from 7,462 journals where significant underrepresentation of LMIC and Female authors was demonstrated. These findings are pervasive and show no positive correlation with the Journal's Impact Factor.

The systematic collection of the Diversity Factor concept would allow for more detailed analysis, highlight gaps in knowledge, and reflect confidence in the translation of related research. Conversion of this metric to an active pipeline would account for the fact that how we define those most at risk will change over time and quantify responses to particular initiatives. Therefore, continuous measurement of outcomes across groups and those

investigating those outcomes will never lose importance. Moving forward, we encourage further revision and improvement by diverse author groups in order to better refine this concept. These four elements should each be seen as a new way to rank journals rather than a simplified score to be optimized. The systematic collection of these features would allow for more detailed analysis, highlight gaps in knowledge, and reflect confidence in the translation of related research. Conversion of this metric to an active pipeline would account for the fact that how we define those most at risk will change over time and quantify responses to particular initiatives. Therefore, the salient point is that continuous measurement of outcomes across groups and those investigating those outcomes will never lose importance.

Background

The last decade has seen our capacity to store, analyse and distribute health data grow exponentially, especially with the growing use of artificial intelligence (AI), yet healthcare has tried and failed to implement it in a successful manner. The current AI landscape is ever-expanding and many of the current models are either still in the prototype stage [1,2] or exhibit substandard performance, particularly in the cases of sepsis and COVID-19 [3,4]. More worryingly, AI has inherent bias, introduced by both the data and those who created it, and it is no surprise that it can disproportionately affect minorities [5,6]. yet the digital health landscape has failed to bridge the gap to implementation (1,2). Furthermore, decision support systems that have been deployed have often resulted in performance significantly below what has been demonstrated in the training sets (3,4). However, more worryingly, the harm being introduced by such algorithms is not felt equally, with minorities shouldering a disproportionate amount of the burden (5). This has led to the call for greater transparency in the model development phase, improved data sharing, and more diversity among research groups to safeguard against such biases [7] (6). However, these changes have not yet reached the journal-level as the current metrics used to evaluate research and journal impact do not focus

Commented [JG1]: Go through and change references to match justin doc

on such factors. Furthermore, the However, current metrics used to evaluate research and journal impact do not focus on such factors, and their ability to provide a complete measure of health research's significance, penetrance, and relevance has been debated for decades [8] (7).

Initially designed to track citations of articles by authors and journals, the impact factor (IF) used to judge the importance of scientific or academic publications and the journal itself. mapped the number of papers published each year and was used to judge the importance of scientific or academic publications and the journal. Though the IF accounted accounts for variations in publishing volumes between journals, the impact on the population or a community had little relevance to the indices [9–12](8–11). Currently, the IF has transformed into a proxy for the quality of individual articles even though highly cited papers skew calculations. As a result, journal figures do not represent the majority of papers in a specific journal [13–16], so journal figures do not represent the majority of papers in a specific journal (12–15). In addition, the IF has several limitations, such as not accounting for the citation density of fields or fluctuations in publication practices, for example, during the COVID-19 pandemic, which inflated critical care journals' impact factors [17,18] (16–18). Moreover, leading academic journals have gamed the system to improve their own IF through self-citation, a practice which is equally common among leading authors [19,20](19,20).

Despite the emphasis on IF, and citations as proxy, they It is important to note that citations do not equate to scientific excellence or the advancement of health research that improves outcomes for all [21](21). There is increasing evidence of disparities in outcomes across demographic groups, where the COVID-19 pandemic was a prime example [22,23](5,22). These disparities are further reinforced by nonrepresentative research, in regards to the lack of diversity in both researchers and questions [24]. Having a research group that better represents their population in question allows for better coverage of multiple problem-solving styles and a better understanding of the problems they face [25,26]. Furthermore, increased

ethnic diversity has been found to be strongly correlated with scientific impact and can help lead to better science [27].

Yet, despite the heavy emphasis on the importance of diversity, there is no objective measure for tracking progress towards inclusivity in science or for evaluating who contributes to health research [7,28]. A shift from a single citation-based metric to using several different metrics that provide a more complete perspective on factors aligned with scientific excellence based on contribution to advancing inclusivity and diversity, improving health outcomes, and achieving equity is therefore necessary.~~The imbalance in the knowledge landscape has reinforced these differences through different numbers of participants, researchers and leaders from each group (23). Currently, there are no objective measures for evaluating who contributes to health research or tracking our progress towards inclusivity in science, even though journals have repeatedly emphasized the importance of diversity (6,24). These limitations demand a shift from citation-based metrics to metrics aligned with scientific excellence based on contribution to advancing inclusivity and diversity, improving health outcomes and achieving equity. Therefore, we introduce a new metric based on key elements that seeks to achieve this goal- the Diversity Factor (DF).~~

Characteristics of a Diversity Factor for assessing Journal Impact

This proposed Diversity Factor (DF) should provide an alternative means of tracking accurate and reliable contributions to health research aligned with the impact on the global community or population in addition to offering an approach to facilitate scientific excellence that is unbiased, representative, and impactful. Important factors that should be considered in evaluating the literature include features related to Publications, Authorship, and Research Oversight. These are explored in Table 1 along with guiding questions that describe the feature's respective characteristics. It is important to note that this is merely a proposal and

should serve as a foundation to build upon. Additionally, this proposed Diversity Factor should serve as a supplement to the currently used metrics and not as a replacement. Scoring of this concept is also left for future revisions. For this paper, each key element is reviewed independent of the others.~~The Diversity Factor aims to propose an alternative means of tracking accurate and reliable contributions to health research aligned with the impact on the global community or population. It offers an approach to facilitate scientific excellence that is unbiased, representative and impactful. Important factors that should be considered in evaluating the literature produced from a given journal are explored in Table 1. These include features related to Publications, Authorship and Research Oversight, and are accompanied by guiding questions that describe a feature's characteristics.~~

In this paper, the authors focused on developing tools that allow for the evaluation of the current landscape in regard to ~~To characterise the current landscape, in this paper, we focussed on developing tools that permit the evaluation of~~ four key elements: dataset properties, author country (LMIC status), author gender (M/F) and organisational affiliation (including multidisciplinary team approach).

Formatted: Not Highlight

1. Dataset Characteristics

Data selection is an inevitable component of research; not all data can be captured and instead strict selection is necessary to answer the research question at hand. However, this inherently creates a restricted view that affects the conclusions drawn, irrespective of domain. The characteristics of datasets used to inform clinical decisions are vital as these conclusions will likely have the greatest relevance to the populations studied. This can be further exacerbated as AI has the potential to perpetuate these biases if left unchecked [29]. The authors propose that datasets used in health research should be mapped to ~~we cannot capture everything and must choose what is measured and where. Strict selection is necessary to answer the research question, but it inherently creates a restricted view that affects the~~

~~conclusions drawn, irrespective of domain. These conclusions will likely have the greatest relevance to the populations studied; therefore, the characteristics of datasets used to inform clinical decisions are vital. Particularly when AI has the potential to perpetuate these biases if left unchecked. We propose that mapping the datasets used in health research can~~ highlight geographic and academic areas of data poverty, expose underlying knowledge gaps, and draw attention to imbalanced datasets~~–~~ [30]~~(25)~~. These include gender imbalance, race-ethnicity, language preference, age, and geography. The current impact factor rewards citations equally but, determining who has read, utilized, and has been 'impacted' by the research is not as simple as implied. Most data being analyzed to guide healthcare is derived from a few centers, almost exclusively based in High-Income Countries [31]. As such, increasing the global impact of research is vital, and the increasing adoption of technology has created the potential to democratize health research. More effort should then be placed on increasing the diversification of the data pool used to design clinical guidelines and develop tools that provide beneficial outcomes for all, and not just a select few countries.~~Increasing the global impact of research is vital, and the increasing adoption of technology has created the potential to democratize health research. Yet, most data being analyzed to guide healthcare is derived from a few centers, almost exclusively based in High-Income Countries (26). Therefore increasing the diversification of the data pool used to design clinical guidelines or develop tools is vital to ensure healthcare can work for all. The current impact factor rewards citations equally. Yet, determining who has read, utilized and been 'impacted' by the research is not as simple as implied.~~

2. Author Country

Author's previous experiences, surrounding culture, and the associated team will significantly shape projects. Thus, when considering how to evaluate an author group, it is important to heavily weigh those with increased diversity, as these groups will better reflect the populations in question as well as having a wider range of problem-solving abilities and perspectives. Evaluating the spread in the country of affiliations within studies can speak to the cognitive

diversity of the teams and the likelihood of methodology and results transferring to that area. The authors designed the study methodology, conducted the analysis, interpreted the findings and presented these in an organized manner. Throughout these stages, biases can be introduced by influencing selection strategies, modes of analyses and presentation of results. Authors working in one country who analyze datasets from another have inherent limitations due to an incomplete understanding of the context and culture of the studied subjects. Including diverse perspectives can maximize the scope for identifying potential biases and ensuring the results produced are applicable to multiple locations. Notably, historical racial prejudices have resulted in disparities in clinical outcomes between demographics and including a variety of ethnicities would improve safeguarding against introducing similar biases [32]. It is commonplace for knowledge to centralize, with intellectual centers producing multitudes of research. These 'Ivory Towers' often overrepresent a particular demographic that is inconsistent with the experience or backgrounds of those most burdened by disease. Increasing diversity within author groups, especially within institutions, can help combat this resulting homogeneity of thought. This is because people often reflect their teachers; therefore, teams solely of Ivory tower academics (ITA) and Taught by ITA can reduce the diversity of thought. Notably, historical racial prejudices have resulted in disparities in clinical outcomes between demographics and including a variety of ethnicities would improve safeguarding against introducing similar biases (27).

3. Author Gender

4. Diversity is more than just increasing the number of ethnicities within the author group. Traditionally, academia has been a male-dominated field, well-documented across multiple fields [33,34][33,34]. However within recent years, this trend has been shifting - more and more women are joining the field. However, gender-parity has not yet been reached and given the current trends will take several more years unless there are active, intentional changes. In this study, an algorithm trained to identify 'gender' as Male or Female was utilized. However,

ormatted: Normal, No bullets or numbering

as further research is done to refine this proposal, expanding this definition to include other genders and the distinction between sex and gender would be ideal.~~Academia has been traditionally conducted by males, although a growing number of women are entering academia, with an associated rising number of Female authors (28). Male and Female physiology and role in society are not the same, and research should not treat it as such. A team with increased female representation would safeguard against introducing practices with sex-based differences in outcomes. It is also important to consider differences in genders which are likely to have different lived experiences, which could also be important for innovation. In this study, we utilized an algorithm trained to identify 'gender' as Male or Female. However, expanding this definition to include other genders and the distinction between sex and gender would also be preferable in the future.~~

~~5.~~4. Organizational or Departmental affiliation

~~6.~~ Most health research has traditionally been conducted in a few institutions with the necessary funding and access to data. Considering the centralization of knowledge and the overrepresentation of certain demographics found in these ivory towers, there has been a recent shift to increase engagement with local stakeholders and increase patient public involvement. Further, healthcare is increasingly becoming a multi-disciplinary field as a result of the recognition that socioeconomic factors play significant roles in health outcomes. As such, m~~Healthcare is increasingly becoming a multi-disciplinary field with socioeconomic factors playing significant roles in health outcomes. M~~ultidisciplinary teams play an important role in bridging professional boundaries and breaking down the barriers of competing cultural and organizational differences will improve the translational gap in medical devices and root academic work in deployable applications. There are currently divides between clinical, academic, and commercial research that often leaves everyone feeling that data is out of reach. Bridging the gap between commercial and non-commercial fields will improve the translational gap in medical devices and root academic

Formatted: Normal, Indent: Left: 0.25", No bullets or numbering

work in deployable applications. Understanding the current breakdown of institutional affiliation, whether it is academic or commercial, is necessary to see if academia is responding to this multidisciplinary call. Expansion of this definition to include the composition of expertise will also be vital, for example, the interaction of machine learning engineers and social scientists in the field of AI.

Analysis of Diversity Factor in Journals with Global Reach

To evaluate the current diversity factor landscape, we used the OpenAlex database consisting of the metadata of all papers published from 2000 until August 2022 [35](29). The entire database was downloaded locally, where metadata, including author name, affiliation, and study abstract, was extracted. Only journals identified by SCImago were included in the analysis.

Dataset Characteristics were not widely named in study abstracts, and due to the lack of freely available full papers, we could not identify this factor reproducibly. The code to implement this feature evaluation has been validated and is freely available for future use [36](30). However, due to these limitations, it has been excluded from the analysis portion of this study.

Author affiliations were identified and geocoded using OpenAlex identification of more than 100,000 research producers in the Research Organisation Registry [37] (31). We geocoded raw affiliation strings for affiliations with no match using a custom Nominatim API [38](32). Geographic locations were matched in 88% of author instances. Thereafter, Author Countries were grouped by income status according to the World Bank; Low-Middle Income Country (LMIC) or non-LMIC Country [39](33).

Enriched metadata is produced using fine-tuned Natural Language Processing models (BERT-Pubmed) for research classification and entity extraction, as described elsewhere

[1]~~(1)~~. Affiliation strings derived from this process were then parsed for information on departmental affiliation that were then categorized into commercial and ~~non-commercial~~academic organizations.

We identified the author's gender using several APIs that demonstrate state-of-the-art performance in validation studies on non-English names, including Gender-API and Genderize [40,41]~~ (34,35)~~. Gender matching was conducted using the first name and affiliation country, with 84% of entries matched. The female: male author ratio was calculated for each paper, and then a mean was calculated for each journal and time period.

Features are displayed individually, and a hypothesis of aggregation is discussed below. Descriptive statistics for each of the three included features is displayed in a subset of eight journals that cover the broadest ranges of speciality, Impact Factor, and traditional prestige. A static dashboard using over 7539 journals was then developed using TableauPublic to represent the full diversity factor landscape and to allow for effect modifiers (open-access and funding sources) to be evaluated; this is available at www.equitablescience.com.

Results

Since the year 2000, 130,721 papers have been identified from 7539 journals, a majority of which are from authors based in North America or Central Asia and Europe. ~~In total, 130,721 papers were identified since 2000 from 7539 journals. There was significant representation from authors based in North America or Central Asia and Europe. ~~In 2021, there were 0.68 and 0.73 authors per paper per region, respectively, compared to under 0.1 in each of Latin America, the Caribbean, South Asia, and Africa. Underrepresentation of female authors is seen throughout. In 2000, median female:male author ratio was 0.40 and has increased over time to 0.78 in 2021. There are a few countries where journals have reached gender parity, such as Portugal (1.13) and Cuba (1.09). However, despite this trend towards greater

Formatted: Not Highlight

representation for female authors, it is unclear how many journals, if any, will reach gender-parity in the next five years given the rate of improvement. Those most at risk of being 'left behind' are primarily from low-income countries though some countries such as Japan (0.24) and South Korea (0.26) face such obstacles as well. Additionally, in 2021, compared to on average. In 2021, the median female: male author ratio was 0.78 and the LMIC: non: LMIC author ratio was 0.014 across all journals.

Authors from low-middle-income countries are sorely underrepresented as well. In 2021, there were over 5 million authors from high-income countries. In comparison, upper-middle-income countries had 1.5 million authors, lower-middle-income countries had 467,323, and low-income countries had 27,080 authors. Across all journals, the LMIC:non-LMIC author ratio was 0.014 in 2021, the result of a slow improvement since 2000 (Figure 1B)

Interestingly, these trends of female author and LMIC author underrepresentation are reversed when looking at a subset of open-access journals. When comparing the top 25 most-impactful open access journals and non-open access journals, open access journals consistently had higher proportions of female authors and authors from LMICs over the past two decades.

Table 2 summarizes the findings from a selection of well-known journals from 2021. The female:male ratio of authors in this subset mirrors the consistent pattern of female author underrepresentation seen in all journals, with none from this subset averaging over 1. Author representation from LMICs is consistently low as well, with most journals under 1:10. In addition, most authors were from academic or non-commercial organizations, highlighting the lack of multidisciplinary collaboration.

Table 2 summarizes findings from a selection of prestigious journals from 2021. The female: male ratio of authors in this subset demonstrates a consistent pattern of female author

underrepresentation, with none of the journal subsets averaging over 1. This is also mirrored when looking at all papers and journals published in the last 21 years, with a general trend towards greater representation, Figure 1A. Despite this, it is unclear whether this trend will see journals reaching total gender parity in the next five years at this trajectory.

Author representation from LMICs is consistently low in this subset and all journals, with a slow trend of improvement (Figure 1B). This subset particularly has a very low proportion of LMIC authors, with most journals under 1:10. In addition, most authors were from academic or non-commercial organizations, highlighting the lack of collaboration even in this prestigious subset.

Discussion

In this paper, the concept of a Diversity Factor was proposed as a supplemental metric we propose a new means of measuring a journal's contribution to the research landscape, focusing on diversity, equity, inclusion, and impact on the studied community or population. Analysis of the data under the lens of the key elements of a proposed Diversity Factor reveal unsurprising results. Female authors and authors from LMICs are sorely underrepresented. While trends are improving for both demographics, several obstacles still stand in the way. Academia is becoming more and more centralized especially in high-income countries. As it does so, it becomes more difficult to penetrate, especially when considering the trends of self-citation.Valuable information can be gathered using study metadata in combination with machine learning tools. There has been a trend towards the ratio of Female: Male authors equalizing in key journals; however, there is still some distance to go if the aim is to reach parity. In contrast, the inclusion of LMIC authors in key journals is consistently low and shows slow improvement across this subset, despite a select few that have increased representation.

Moreover, the current forms of impact are not strong indicators of increased diversity; in contrast, they may be negatively associated.

What steps to take in order to help improve inclusivity and diversity lie outside the scope of this paper. However, the reversal of trends seen when comparing open-access journals to non-open-access journals may be an angle worth further investigation. These results are most likely due to the easier accessibility found with the open-access model but are worth noting.

The heterogeneity in the findings between diversity metrics and between journals likely means that these four values should not be combined into one 'diversity value'. Instead, it should represent four components that should be evaluated and compared individually, see Figure 2. Although more complex, it would be favorable to use star diagrams or visual tools to represent this data. Seeing each metric as a new way to rank journals rather than a simplified score to be optimized. We propose having such values measured at least yearly to offer checkpoints to compare against the traditional impact factor. We have presented these findings and a more detailed analysis from this paper online to permit comparisons across open access, funding sources and specific use cases such as COVID-19 (36). Moreover, our code is freely available online in the interest of reproducibility, the addition of other features, and conversion to an active pipeline (30).

The next steps for implementing a diversity factor would include the availability of dataset characteristics, detailed funding sources, patents and downstream policy impact, and citation mapping. This would allow for a better understanding of who is impacted and who is causing the impact. Additionally, refining the use of author country to a place of birth or time spent in a country would account for those in LMICs who emigrate to other institutions, which is not uncommon. Further, the improvement of gender data or NLP algorithms to account for author gender compared to sex would be another step forward. The operationalisation of this tool would rely on interest from journals and researchers in collaborating to permit this data to be published on each journal's website and centrally for evaluation. The heterogeneity in the

findings between these four diversity metrics and between journals likely means that these four values should not be combined into one ‘diversity value’. Instead, they should be evaluated and compared individually as seen in Figure 2, or other visual tools such as in a star diagram.

The next steps for the diversity factor would include the availability of dataset characteristics, detailed funding sources, patents and downstream policy impact, and citation mapping. This would further the goal of measuring who is impacted and who is making the impact. Additionally, refining the use of author country to a place of birth or time spent in a country would account for those in LMICs who emigrate to other institutions, which is not uncommon. Further, the improvement of gender data or NLP algorithms to account for author gender compared to sex would be another step forward. The operationalisation of this tool would rely on interest from journals and researchers in collaborating to permit this data to be published on each journal's website and centrally for evaluation.

Additionally, we propose having such values rechecked yearly to offer checkpoints and track progress as new research is published and populations evolve. Routine reporting would further allow for more detailed analysis to be performed, highlight gaps in knowledge, and reflect confidence in the translation of related research. The routine reporting of features included in the DF, particularly the dataset characteristics, would allow for more detailed analysis to be performed, highlight gaps in knowledge, and reflect confidence in the translation of related research. This is particularly true at the health policy level, where it is known that the social determinants of health vary greatly between countries; therefore, clinical decisions and public health decisions should be made based on information more representative of these populations. It is important to acknowledge that there is not one type of bias, nor one group affected by bias, but many types of bias and many groups that can be biased against. Diversity is not a box-ticking exercise but an essential safeguard against potential biases, especially in countries with greater ethnicity, cultural diversity and particular socio-demographic characteristics.

Formatted: Not Highlight

Promotion of these features would encourage thoughtful discourse on how study designs and data characteristics can affect different groups so that researchers and organizations can build the right team for the specific project and its risks. Furthermore, integration of the Diversity Factor can encourage collaboration with LMICs that could reshape the knowledge landscape through the dissemination of work partnered with LMICs.

~~Conversion of this metric to an active pipeline would account for the fact that how we define those who are most at risk will change over time. This approach would facilitate the quantification of representation and measurement in response to particular initiatives. Therefore, the salient point is that continuous measurement of outcomes across groups and those investigating those outcomes will never lose importance. Systems must be built and regularly updated to protect and serve those at risk of discrimination—the diversity factor is our attempt to step towards this measurement.~~

Our goal was to evaluate the current diversity factor landscape to the highest degree permissible by the availability of data and the current standards of metadata. Data set characteristics were not widely available, which did not permit the evaluation of this important feature. However, we have provided the tools to implement this feature if made available in the future. Gender was determined using NLP, which has demonstrated good performance across countries; however, the ground truth is not available for these studies and so cannot be confirmed. SCImago Journal Ranking is used in place of traditional impact factor due to the open source nature and availability of the feature, there is a similarity between the two, but we also acknowledge differences. We recognise these tools are imperfect, but we hope they provide a ‘big picture’ view of the global research landscape and demonstrate what is possible in the future with greater open access to journals and why these metrics could greatly promote the drive for equitable science.

The four elements used in this paper uncovers a bleak reality unseen by citation-based metrics. Academia as it is now, and the healthcare systems that it shapes, cannot equitably and justly provide for all if it is not reflective of the populations in question. And in order to do that, it is necessary to change the way science evaluates its efforts. The current The currently available methods of measuring journal impact are far from ideal and fail to provide an estimate of author and dataset diversity. While diversity should not be the only consideration of researchers or journals, it should complement the downstream citation impact. Yet, tracking who participates in the conversations that shape healthcare and where our opinions are being formed should be monitored and evaluated transparently and publicly.

The Diversity Factor is a call to action for improved representation and the encouragement of diverse perspectives in health research to prevent the perpetuation of biases against subgroups and the advancement of scientific excellence that works for all. It reminds journals and authors to assess how thoughts and data reach the manuscript and whether they consider all perspectives, not just those available at hand. Otherwise, we will continue learning and practising medicine in an echo chamber created by the few Ivory Tower Academics with access to the resources and data required to advance the field left in the hands of a select few institutions.

These findings and a more detailed analysis from this paper are available online to permit comparisons across open access, funding sources and specific use cases such as COVID-19 [42]. Moreover, the code is also freely available online in the interest of reproducibility, the addition of other features, and for later conversion to an active pipeline [36].

Acknowledgements

We would like to acknowledge the contribution of the OpenAlex database, without which this project would not have been possible.

Author Contributions

Conceptualization, JG, JZ, and LC.; Methodology and Formal Analysis, JG, JZ, SW and LC; Writing – Original Draft, JG and JZ; Writing – Review & Editing, All authors.

Declaration of interests

The authors declare no conflicts of interest.

Figure and scheme legends

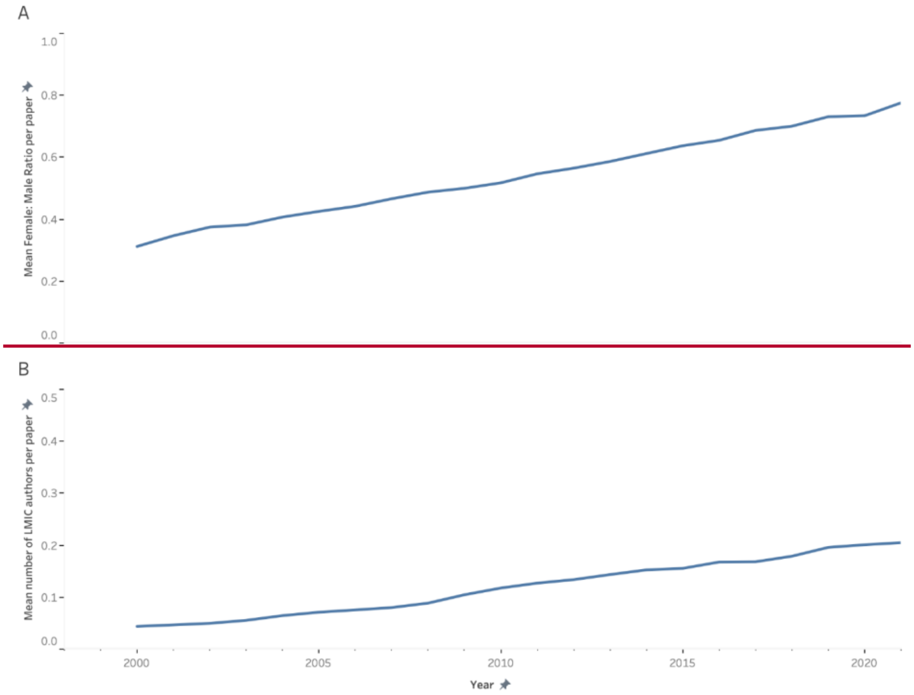

Figure 1. The mean value for **A)** median Female: Male author ratio on a paper per journal each year from 2000-2021, **B)** median LMIC: non-LMIC author ratio per journal each year from 2000-2021.

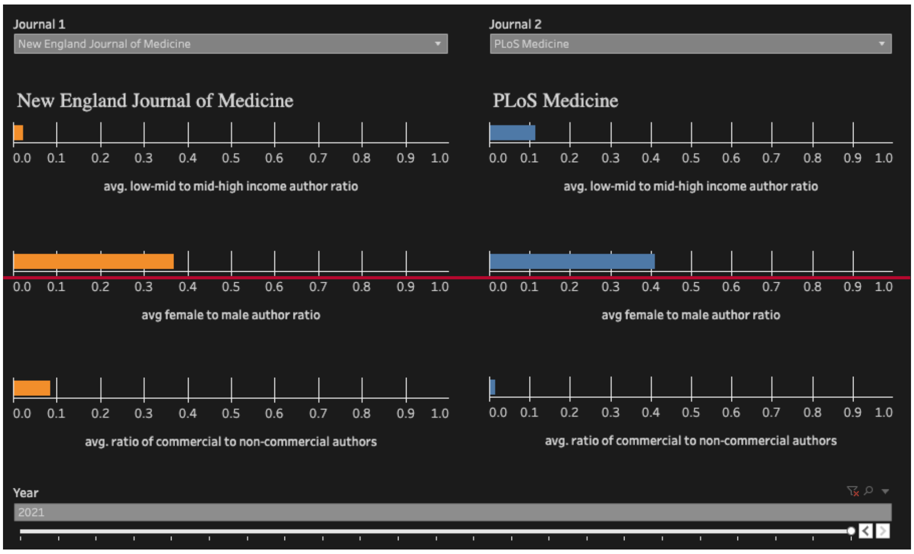

Figure 2. Comparison of the individual features of the Diversity factor for two journals, from https://equitablescience.com/dashboards/diversity/.

Tables

Formatted: Not Highlight

Table 1. Elements for Assessing Journal Contribution to Scientific Excellence in Diversity, Equity and Inclusion.

Formatted: Not Highlight

**Category**	**Element**	**Guiding Questions**
**Publications**	Diversity	Do studies explore health, determinants of health and underlying factors driving improved outcomes for diverse population groups?
	Equity	Do publications showcase research relevant to under-represented communities and populations?
	Inclusion	Have publications promoted inclusive and multidisciplinary research methods?
	Datasets	What are the definitions and distributions of age, race, ethnicity, gender, language, and geography, included in the study participants?
**Authorship**	Author identity, country or origin	Is there a wide range of author cultures, experience and expertise, including country or origin and low-middle income countries (LMICs)?
	Author Gender	Is there a balanced gender distribution on average among authors, and first/last authors?

	Author Organization and Affiliation	Is there a diverse range of organizations, including minority serving institutions, industry and academia?
	Community experts	Has the published research been conducted with community experts, and are they named co-authors?
**Research Oversight**	Journal Editors and Reviewers	Has the journal developed an accountability system to measure and ensure diversity among editors and reviewers?
	Review Process	Does a journal have a system to identify and respond to potential bias in the review process?
	Communication and Dissemination	Are the results of the above questions easily accessible and transparent for researchers and the general public to review?

Formatted: Not Highlight

Table 2. Comparison of median diversity factors across journals in 2021

Journal	SJR (IF)	Female: Male *	LMIC: non-LMIC author *	Commercial: Non-commercial affil.*
BMJ	2291 (93)	0.43	0.03	0.02
JAMA	6076 (157)	0.40	0.01	0.03
Lancet	6024 (202)	0.37	0.15	0.03
Nature Medicine	15652 (87)	0.38	0.02	0.05
NEJM	24161 (176)	0.36	0.03	0.09
PLoS Medicine	24907 (12)	0.42	0.12	0.01
npj Digital Medicine	4180 (15)	0.30	0.02	0.12
The Lancet. Digital health	3326 (37)	0.31	0.03	0.05

SJR= Scimago Journal Ranking, Average number of weighted citations received in a year by articles published in a journal in the year 2021. IF = Impact Factor. * Ratio of mean values per paper for each journal 2dp. Commercial affil.= the mean number of authors per paper from a commercial company.

References

1. Zhang J, Whebell S, Gallifant J, Budhdeo S, Mattie H, Lertvittayakumjorn P, et al. An interactive dashboard to track themes, development maturity, and global equity in clinical artificial intelligence research. The Lancet Digital Health. 2022;4: e212–e213. doi:10.1016/S2589-7500(22)00032-2

2. van de Sande D, van Genderen ME, Huiskens J, Gommers D, van Bommel J. Moving from bytes to bedside: a systematic review on the use of artificial intelligence in the intensive care unit. Intensive Care Med. 2021;47: 750–760. doi:10.1007/s00134-021-06446-7

3. Wong A-KI, Charpignon M, Kim H, Josef C, de Hond AAH, Fojas JJ, et al. Analysis of Discrepancies Between Pulse Oximetry and Arterial Oxygen Saturation Measurements by Race and Ethnicity and Association With Organ Dysfunction and Mortality. JAMA Netw Open. 2021;4: e2131674–e2131674. doi:10.1001/jamanetworkopen.2021.31674

4. Singh K, Valley TS, Tang S, Li BY, Kamran F, Sjoding MW, et al. Evaluating a Widely Implemented Proprietary Deterioration Index Model among Hospitalized Patients with COVID-19. Ann Am Thorac Soc. 2021;18: 1129–1137. doi:10.1513/AnnalsATS.202006-698OC

5. HealthLeaders. Root of some health disparities may be buried in technology. [cited 30 Nov 2022]. Available: https://www.healthleadersmedia.com/technology/root-some-health-disparities-may-be-buried-technology

6. Gianfrancesco MA, Tamang S, Yazdany J, Schmajuk G. Potential Biases in Machine Learning Algorithms Using Electronic Health Record Data. JAMA Intern Med. 2018;178: 1544–1547. doi:10.1001/jamainternmed.2018.3763

7. Oh SS, Galanter J, Thakur N, Pino-Yanes M, Barcelo NE, White MJ, et al. Diversity in Clinical and Biomedical Research: A Promise Yet to Be Fulfilled. PLoS Med. 2015;12: e1001918. doi:10.1371/journal.pmed.1001918

8. Singh Chawla D. What’s wrong with the journal impact factor in 5 graphs. nature index; 2018. Available: https://www.nature.com/nature-index/news-blog/whats-wrong-with-the-jif-in-five-graphs

9. Garfield E. Citation Analysis as a Tool in Journal Evaluation. Science. 1972;178: 471–479. doi:10.1126/science.178.4060.471

10. Garfield E, Sher I. Genetics Citation Index. Annu Rep Can Inst Sci Tech Inf/Rapp Annu Inst Can Inf Sci Tech. 1963. Available: http://www.garfield.library.upenn.edu/essays/v7p515y1984.pdf

11. Brodman E. Choosing Physiology Journals. Bull Med Libr Assoc. 1944;32: 479–483.

12. Garfield E. Citation Analysis as a Tool in Journal Evaluation. Science. 1972;178: 471–479. doi:10.1126/science.178.4060.471

13. Bornmann L, Marx W. How good is research really? EMBO Rep. 2013;14: 226–230. doi:10.1038/embor.2013.9

14. Paulus FM, Cruz N, Krach S. The Impact Factor Fallacy. Front Psychol. 2018;9. doi:10.3389/fpsyg.2018.01487

15. Bornmann L, Marx W, Gasparyan AY, Kitas GD. Diversity, value and limitations of the journal impact factor and alternative metrics. Rheumatol Int. 2012;32: 1861–1867. doi:10.1007/s00296-011-2276-1

16. Dimitrov JD, Kaveri SV, Bayry J. Metrics: journal's impact factor skewed by a single paper. Nature. 2010;466: 179–179. doi:10.1038/466179b

17. Di Girolamo N, Meursinge Reynders R. Characteristics of scientific articles on COVID-19 published during the initial 3 months of the pandemic. Scientometrics. 2020;125: 795–812. doi:10.1007/s11192-020-03632-0

18. Fraser N, Brierley L, Dey G, Polka JK, Pálfy M, Nanni F, et al. The evolving role of preprints in the dissemination of COVID-19 research and their impact on the science communication landscape. PLoS Biol. 2021;19: e3000959. doi:10.1371/journal.pbio.3000959

19. Mishra S, Fegley BD, Diesner J, Torvik VI. Self-citation is the hallmark of productive authors, of any gender. PLoS One. 2018;13: e0195773. doi:10.1371/journal.pone.0195773

20. Taşkın Z, Doğan G, Kulczycki E, Zuccala AA. Self-Citation Patterns of Journals Indexed in the Journal Citation Reports. J Informetr. 2021;15: 101221. doi:10.1016/j.joi.2021.101221

21. Ray KS, Zurn P, Dworkin JD, Bassett DS, Resnik DB. Citation bias, diversity, and ethics. Account Res. 2022; 1–15. doi:10.1080/08989621.2022.2111257

22. Sze S, Pan D, Nevill CR, Gray LJ, Martin CA, Nazareth J, et al. Ethnicity and clinical outcomes in COVID-19: A systematic review and meta-analysis. eClinicalMedicine. 2020;29. doi:10.1016/j.eclinm.2020.100630

23. Magesh S, John D, Li WT, Li Y, Mattingly-app A, Jain S, et al. Disparities in COVID-19 Outcomes by Race, Ethnicity, and Socioeconomic Status: A Systematic Review and Meta-analysis. JAMA Network Open. 2021;4: e2134147–e2134147. doi:10.1001/jamanetworkopen.2021.34147

24. Seastedt KP, Schwab P, O'Brien Z, Wakida E, Herrera K, Marcelo PGF, et al. Global healthcare fairness: We should be sharing more, not less, data. PLOS Digital Health. 2022;1: e0000102. doi:10.1371/journal.pdig.0000102

25. Aminpour P, Schwermer H, Gray S. Do social identity and cognitive diversity correlate in environmental stakeholders? A novel approach to measuring cognitive distance within and between groups. PLOS ONE. 2021;16: e0244907. doi:10.1371/journal.pone.0244907

26. Meissner P, Wulf T. The effect of cognitive diversity on the illusion of control bias in strategic decisions: An experimental investigation. European Management Journal. 2017;35: 430–439. doi:10.1016/j.emj.2016.12.004

27. AlShebli BK, Rahwan T, Woon WL. The preeminence of ethnic diversity in scientific collaboration. Nat Commun. 2018;9: 5163. doi:10.1038/s41467-018-07634-8

28. Striving for Diversity in Research Studies. N Engl J Med. 2021;385: 1429–1430. doi:10.1056/NEJMe2114651

29. Panch T, Mattie H, Atun R. Artificial intelligence and algorithmic bias: implications for health systems. J Glob Health. 2019;9: 010318. doi:10.7189/jogh.09.020318

30. Ibrahim H, Liu X, Zariffa N, Morris AD, Denniston AK. Health data poverty: an assailable barrier to equitable digital health care. The Lancet Digital Health. 2021;3: e260–e265. doi:10.1016/S2589-7500(20)30317-4

31. Smyth B, Trongtrakul K, Haber A, Talbot B, Hawley C, Perkovic V, et al. Inequities in the global representation of sites participating in large, multicentre dialysis trials: a systematic review. BMJ Global Health. 2019;4: e001940. doi:10.1136/bmjgh-2019-001940

32. Cerdeña JP, Plaisime MV, Tsai J. From race-based to race-conscious medicine: how anti-racist uprisings call us to act. Lancet. 2020;396: 1125–1128. doi:10.1016/S0140-6736(20)32076-6

33. Vincent J-L, Juffermans NP, Burns KEA, Ranieri VM, Pourzitaki C, Rubulotta F. Addressing gender imbalance in intensive care. Critical Care. 2021;25: 147. doi:10.1186/s13054-021-03569-7

34. The Lack of Diversity in Healthcare on JSTOR. [cited 14 May 2023]. Available: https://www.jstor.org/stable/26894210

35. Priem J, Piwowar H, Orr R. OpenAlex: A fully-open index of scholarly works, authors, venues, institutions, and concepts. 2022; arXiv:2205.01833.

36. whizzlab. health_ai_question-answer. 2022 [cited 1 Sep 2022]. Available: https://github.com/whizzlab/health_ai_question_answer

37. Research Organization Registry. 2022 [cited 1 Sep 2022]. Available: https://ror.org/

38. Nominatim team. Nominatim. 2022 [cited 1 Sep 2022]. Available: https://nominatim.org/

39. The World Bank. World Bank Open Data,. 2022 [cited 1 Sep 2022]. Available: https://data.worldbank.org/

40. Gender API Team. Gender API. 2022 [cited 1 Sep 2022]. Available: https://gender-api.com/

41. Genderize Team. Genderize API. 2022. Available: https://genderize.io/

42. Equitable Science. 2022 [cited 1 Nov 2022]. Available: https://equitablescience.com/

~~1. Zhang J, Whebell S, Gallifant J, Budhdeo S, Mattie H, Lertvittayakumjorn P, et al. An interactive dashboard to track themes, development maturity, and global equity in clinical artificial intelligence research. The Lancet Digital Health [Internet]. 2022 [cited 2022 Nov 28];4(4):e212–3. Available from: https://doi.org/10.1016/S2589-7500(22)00032-2~~

~~2. van de Sande D, van Genderen ME, Huiskens J, Gommers D, van Bommel J. Moving from bytes to bedside: a systematic review on the use of artificial intelligence in the intensive care unit. Intensive Care Med [Internet]. 2021 Jul;47(7):750–60. Available from: http://dx.doi.org/10.1007/s00134-021-06446-7~~

Formatted: Space Before: 11 pt

3. Wong A, Otles E, Donnelly JP, Krumm A, McCullough J, DeTroyer-Cooley O, et al. External Validation of a Widely Implemented Proprietary Sepsis Prediction Model in Hospitalized Patients. JAMA Intern Med [Internet]. 2021 Aug 1;181(8):1065–70. Available from: http://dx.doi.org/10.1001/jamainternmed.2021.2626

4. Singh K, Valley TS, Tang S, Li BY, Kamran F, Sjoding MW, et al. Evaluating a Widely Implemented Proprietary Deterioration Index Model among Hospitalized Patients with COVID-19. Ann Am Thorac Soc [Internet]. 2021 Jul;18(7):1129–37. Available from: http://dx.doi.org/10.1513/AnnalsATS.202006-698OC

5. Magesh S, John D, Li WT, Li Y, Mattingly-app A, Jain S, et al. Disparities in COVID-19 Outcomes by Race, Ethnicity, and Socioeconomic Status: A Systematic Review and Meta-analysis. JAMA Network Open [Internet]. 2021 [cited 2022 Nov 28];4(11):e2134147–e2134147. Available from: https://doi.org/10.1001/jamanetworkopen.2021.34147

6. Oh SS, Galanter J, Thakur N, Pino-Yanes M, Barcelo NE, White MJ, et al. Diversity in Clinical and Biomedical Research: A Promise Yet to Be Fulfilled. PLoS Med [Internet]. 2015;12(12):e1001918. Available from: https://doi.org/10.1371/journal.pmed.1001918

7. What's wrong with the journal impact factor in 5 graphs [Internet]. Nature Index. [cited 2022 Nov 29]. Available from: https://www.nature.com/nature-index/news-blog/whats-wrong-with-the-jif-in-five-graphs

8. Garfield E. Citation indexes for science; a new dimension in documentation through association of ideas. Science [Internet]. 1955 Jul 15;122(3159):108–11. Available from: http://dx.doi.org/10.1126/science.122.3159.108

9. Institute for Scientific Information. Genetics Citation Index: Experimental Citation Indexes to Genetics with Special Emphasis on Human Genetics [Internet]. 1963. 1044 p. Available from: https://books.google.com/books/about/Genetics_Citation_Index.html?hl=&id=P8Q9AAAAIAAJ

10. Brodman E. Choosing Physiology Journals. Bull Med Libr Assoc [Internet]. 1944 Oct;32(4):479–83. Available from: https://www.ncbi.nlm.nih.gov/pubmed/16016669

11. Garfield E. Citation Analysis as a Tool in Journal Evaluation. Science [Internet]. 1972;178(4060):471–9. Available from: https://www.science.org/doi/abs/10.1126/science.178.4060.471

12. Bornmann L, Marx W. How good is research really? Measuring the citation impact of publications with percentiles increases correct assessments and fair comparisons. EMBO Rep [Internet]. 2013 Mar 1;14(3):226–30. Available from: http://dx.doi.org/10.1038/embor.2013.9

13. Paulus FM, Cruz N, Krach S. The Impact Factor Fallacy. Front Psychol [Internet]. 2018 Aug 20;9. Available from: https://www.frontiersin.org/articles/10.3389/fpsyg.2018.01487

14. Bornmann L, Marx W, Gasparyan AY, Kitas GD. Diversity, value and limitations of the journal impact factor and alternative metrics. Rheumatol Int [Internet]. 2012 Jul 1;32(7):1861–7. Available from: https://doi.org/10.1007/s00296-011-2276-1

15. Dimitrov JD, Kaveri SV, Bayry J. Metrics: journal's impact factor skewed by a single paper. Nature [Internet]. 2010 Jul 8;466(7303):179. Available from: http://dx.doi.org/10.1038/466179b

16. Di Girolamo N, Meursinge Reynders R. Characteristics of scientific articles on COVID-19 published during the initial 3 months of the pandemic. Scientometrics [Internet]. 2020 Oct 1;125(1):795–812. Available from: https://doi.org/10.1007/s11192-020-03632-0

17. Fraser N, Brierley L, Dey G, Polka JK, Pálfy M, Nanni F, et al. The evolving role of preprints in the dissemination of COVID-19 research and their impact on the science communication landscape. PLoS Biol [Internet]. 2021;19(4):e3000959. Available from: https://doi.org/10.1371/journal.pbio.3000959

18. Garfield E. The History and Meaning of the Journal Impact Factor. JAMA [Internet]. 2006 [cited 2022 Nov 28];295(1):90–3. Available from: https://doi.org/10.1001/jama.295.1.90

19. Mishra S, Fegley BD, Diesner J, Torvik VI. Self-citation is the hallmark of productive authors, of any gender. PLoS One [Internet]. 2018;13(9):e0195773. Available from: https://doi.org/10.1371/journal.pone.0195773

20. Taşkın Z, Doğan G, Kulczycki E, Zuccala AA. Self-Citation Patterns of Journals Indexed in the Journal Citation Reports. J Informetr [Internet]. 2021 Nov 1;15(4):101221. Available from: https://www.sciencedirect.com/science/article/pii/S1751157721000924

21. Ray KS, Zurn P, Dworkin JD, Bassett DS, Resnik DB. Citation bias, diversity, and ethics. Account Res [Internet]. 2022;1–15. Available from: https://doi.org/10.1080/08989621.2022.2111257

22. Sze S, Pan D, Nevill CR, Gray LJ, Martin CA, Nazareth J, et al. Ethnicity and clinical outcomes in COVID-19: A systematic review and meta-analysis. eClinicalMedicine [Internet]. 2020 [cited 2022 Nov 28];29. Available from: https://doi.org/10.1016/j.eclinm.2020.100630

23. Seastedt KP, Schwab P, O'Brien Z, Wakida E, Herrera K, Marcelo PGF, et al. Global healthcare fairness: We should be sharing more, not less, data. PLOS Digital Health [Internet]. 2022;1(10):e0000102. Available from: https://doi.org/10.1371/journal.pdig.0000102

24. Striving for Diversity in Research Studies. N Engl J Med [Internet]. 2021;385(15):1429–30. Available from: https://www.nejm.org/doi/full/10.1056/NEJMe2114651

25. Ibrahim H, Liu X, Zariffa N, Morris AD, Denniston AK. Health data poverty: an assailable barrier to equitable digital health care. The Lancet Digital Health [Internet]. 2021 [cited 2022 Nov 28];3(4):e260–5. Available from: https://doi.org/10.1016/S2589-7500(20)30317-4

26. Smyth B, Trongtrakul K, Haber A, Talbot B, Hawley C, Perkovic V, et al. Inequities in the global representation of sites participating in large, multicentre dialysis trials: a systematic review. BMJ Global Health [Internet]. 2019;4(6):e001940. Available from: https://gh.bmj.com/content/bmjgh/4/6/e001940.full.pdf

27. Cerdeña JP, Plaisime MV, Tsai J. From race-based to race-conscious medicine: how anti-racist uprisings call us to act. Lancet [Internet]. 2020 [cited 2022 Nov 28];396(10257):1125–8. Available from: https://doi.org/10.1016/S0140-6736(20)32076-6

28. Chen JT, Qadir N, Hope AA, Gershengorn HB. Shining a Light on Diversity in Team Science*. Crit Care Med [Internet]. 2022;50(4):685–7. Available from: https://journals.lww.com/ccmjournal/Fulltext/2022/04000/Shining_a_Light_on_Diversity_in_Team_Science_.16.aspx

29. Priem J, Piwowar H, Orr R. OpenAlex: A fully-open index of scholarly works, authors, venues, institutions, and concepts. 2022 May 1;arXiv:2205.01833. Available from: https://ui.adsabs.harvard.edu/abs/2022arXiv220501833P

30. whizzlab. health_ai_question-answer [Internet]. 2022 [cited 2022 Sep]. Available from: https://github.com/whizzlab/health_ai_question_answer

31. Research Organization Registry,. 2022 [cited 2022 Sep]. Available from: https://ror.org/

32. Nominatim team,. Nominatim [Internet]. 2022 [cited 2022 Sep]. Available from: https://nominatim.org/

33. The World Bank. World Bank Open Data, [Internet]. 2022 [cited 2022 Sep]. Available from: https://data.worldbank.org/

34. Gender API Team,. Gender API [Internet]. 2022 [cited 2022 Sep]. Available from: https://gender-api.com/

35. Genderize Team,. Genderize API [Internet]. 2022. Available from: https://genderize.io/

36. Equitable Science [Internet]. 2022 [cited 2022 Nov]. Available from: https://equitablescience.com/

Dear Editor,

Thank you for the opportunity to submit a revision of our paper on the Diversity Factor. We are pleased that you have been very supportive of its publication in PLOS Global Public Health.

We have made the revisions as suggested and are grateful for your time and critical insight, which has improved the study further.

Thank you for handling our submission, and we look forward to hearing from the journal soon.

Yours sincerely

We thank the Editor and Reviewer for their constructive thoughts on this piece. The paper has been rewritten and restructured to strengthen the argument as suggested. Concepts were expanded upon as necessary.

[Issue 1]: The manuscript requires an overall strengthening of the argument. For example, concepts such as commercial and non-commercial fields, ITA, etc., can be better explained and expanded on.
The paper has been rewritten and restructured to strengthen the argument as suggested. Concepts were expanded upon as necessary. Changes are highlighted in the document for clarity.

[Issue 2]: The literature review needs to be expanded to include more recent and relevant studies and a more comprehensive analysis of the existing body of research.
Literature review was expanded as requested. 8 additional references were added.

[Issue 3]: The connection between author diversity and research impact needs to be clearly defined.The manuscript would strongly benefit from a more robust and nuanced explanation of this connection.
The entire paper was rewritten to explain this connection better throughout. This is best seen in Lines 120-124.

[Issue 4]: The connection of the diversity factor and the field of digital health needs to be described in more detail as it currently leads to more questions than answers. Please review this by either highlighting this connection or by avoiding using the term digital health, as it entails a specific definition of a particular field.
This is a very important clarification to make. We have elaborated on this throughout the paper to highlight the necessity of the importance of diversity factor; the term digital health was also removed.

[Issue 5]: Line 158, cognitive diversity does not necessarily convey the diversity of expertise, lived experiences, or points of view and I encourage you to revisit this phrase/concept.

Now starts at line 172. This was rewritten to better explain the importance of diversity as a whole.

[Issue 6]: Line 175, 176, there is an underlying assumption that having female authors (better to use gender terms instead of sex terms, i.e., women authors) would automatically reduce the bias and safeguard against inequitable practices. However, this does not necessarily hold as both men and women operate within a patriarchal culture and system and one's sex and/or gender does not automatically create immunity against and departure from patriarchal practices.
This topic is now at lines 193-200. This was rewritten to remove this assumption.

[Issue 7]: Line 231, 232 reads "Additionally, in 2021, compared to on average."
Thank you for catching this error, this has been removed from the manuscript.

[Issue 8]: Lines 244 to 246, it is unclear what kind of collaboration would improve the impact, if that applies to all types of research mentioned in this manuscript, how collaboration is defined and implemented. This concept of collaboration can benefit from further exploration and elaboration.
This was rewritten to emphasize multidisciplinary collaboration. This is now at line 285.

[Issue 9]: Using phrases such as “prestigious subset” in lines 245, 246 creates a tone that does not necessarily go along with the transformative ideas behind the manuscript.
The modifier “prestigious” was removed. This section is now tackled in lines 280-285.

Reviewer #1 Comments
‘This is mentioned in the abstract after mentioning the “diversity factor” – “Herein, we develop the methodology to reproducibly calculate these elements, evaluate the current landscape and publish the findings on a freely available website that enables further evaluation”. However, this sentence failed to establish consistency and its relationship with the diversity factor. Hence, some restructuring is advised

The authors should have described how the diversity factor would be scored which can help the readers to visualize and compare the factor between journals’
The paper has been rewritten to better explain this. In regards to diversity factor scoring, this is addressed in Lines 66-68 in the Abstract and later in Lines 141-144 and 310-314.
