# Supplementary material for: A new tool for evaluating health equity in academic journals; the Diversity Factor
Source: PLOS Glob Public Health. 2023 Aug 14;3(8):e0002252. doi: 10.1371/journal.pgph.0002252 (PMC10424852; doi:10.1371/journal.pgph.0002252)
Supplement: Attachment — Submitted filename: Response to Reviewers.docx [file pgph.0002252.s001.docx]

Dear Editor,

Thank you for the opportunity to submit a revision of our paper on the Diversity Factor. We are

pleased that you have been very supportive of its publication in PLOS Global Public Health.

We have made the revisions as suggested and are grateful for your time and critical insight, which has improved the study further.

Thank you for handling our submission, and we look forward to hearing from the journal soon.

Yours sincerely

We thank the Editor and Reviewer for their constructive thoughts on this piece. The paper has been rewritten and restructured to strengthen the argument as suggested. Concepts were expanded upon as necessary.

[Issue 1]: The manuscript requires an overall strengthening of the argument. For example, concepts such as commercial and non-commercial fields, ITA, etc., can be better explained and expanded on.

The paper has been rewritten and restructured to strengthen the argument as suggested. Concepts were expanded upon as necessary. Changes are highlighted in the document for clarity.

[Issue 2]: The literature review needs to be expanded to include more recent and relevant studies and a more comprehensive analysis of the existing body of research.

Literature review was expanded as requested. 8 additional references were added.

[Issue 3]: The connection between author diversity and research impact needs to be clearly defined.The manuscript would strongly benefit from a more robust and nuanced explanation of this connection.

The entire paper was rewritten to explain this connection better throughout. This is best seen in Lines 120-124.

[Issue 4]: The connection of the diversity factor and the field of digital health needs to be described in more detail as it currently leads to more questions than answers. Please review this by either highlighting this connection or by avoiding using the term digital health, as it entails a specific definition of a particular field.

This is a very important clarification to make. We have elaborated on this throughout the paper to highlight the necessity of the importance of diversity factor; the term digital health was also removed.

[Issue 5]: Line 158, cognitive diversity does not necessarily convey the diversity of expertise, lived experiences, or points of view and I encourage you to revisit this phrase/concept.

Now starts at line 172. This was rewritten to better explain the importance of diversity as a whole.

[Issue 6]: Line 175, 176, there is an underlying assumption that having female authors (better to use gender terms instead of sex terms, i.e., women authors) would automatically reduce the bias and safeguard against inequitable practices. However, this does not necessarily hold as both men and women operate within a patriarchal culture and system and one&#39;s sex and/or gender does not automatically create immunity against and departure from patriarchal practices.

This topic is now at lines 193-200. This was rewritten to remove this assumption.

[Issue 7]: Line 231, 232 reads &quot;Additionally, in 2021, compared to on average.&quot;

Thank you for catching this error, this has been removed from the manuscript.

[Issue 8]: Lines 244 to 246, it is unclear what kind of collaboration would improve the impact, if that applies to all types of research mentioned in this manuscript, how collaboration is defined and implemented. This concept of collaboration can benefit from further exploration and elaboration.

This was rewritten to emphasize multidisciplinary collaboration. This is now at line 285.

[Issue 9]: Using phrases such as “prestigious subset” in lines 245, 246 creates a tone that does not necessarily go along with the transformative ideas behind the manuscript.

The modifier “prestigious” was removed. This section is now tackled in lines 280-285.

**Reviewer #1 Comments**

‘This is mentioned in the abstract after mentioning the “diversity factor” – “Herein, we develop the methodology to reproducibly calculate these elements, evaluate the current landscape and publish the findings on a freely available website that enables further evaluation”. However, this sentence failed to establish consistency and its relationship with the diversity factor. Hence, some restructuring is advised

The authors should have described how the diversity factor would be scored which can help the

readers to visualize and compare the factor between journals’

The paper has been rewritten to better explain this. In regards to diversity factor scoring, this is addressed in Lines 66-68 in the Abstract and later in Lines 141-144 and 310-314.
