# Supplementary material for: A new tool for evaluating health equity in academic journals; the Diversity Factor
Source: PLOS Glob Public Health. 2023 Aug 14;3(8):e0002252. doi: 10.1371/journal.pgph.0002252 (PMC10424852; doi:10.1371/journal.pgph.0002252)
Supplement: Attachment — Submitted filename: final_plos_gh_Response_to_Reviewers.docx [file pgph.0002252.s003.docx]

Dear Editor,

Thank you for your detailed insights that have significantly improved the quality of this manuscript.

We have made all the revisions as per the comments provided, except for one minor comment.

The penultimate comment states ‘publication or dataset?’. We have chosen to use Publication as a header in our table as the dataset forms one component of the publication’s diversity. The methodology of data collection and population engagement are also key factors, as you rightly raise earlier in the text. We are consistent throughout the text when referring to the dataset and when referring to the publication.

Thank you again for all your insight and handling our submission. We look forward to hearing from the journal soon.

Yours sincerely
